# The Putative Roles and Functions of Indel, Repetition and Duplication Events in Alphavirus Non-Structural Protein 3 Hypervariable Domain (nsP3 HVD) in Evolution, Viability and Re-Emergence

**DOI:** 10.3390/v13061021

**Published:** 2021-05-28

**Authors:** Nurshariza Abdullah, Nafees Ahemad, Konstantinos Aliazis, Jasmine Elanie Khairat, Thong Chuan Lee, Siti Aisyah Abdul Ahmad, Nur Amelia Azreen Adnan, Nur Omar Macha, Sharifah Syed Hassan

**Affiliations:** 1 Jeffrey Cheah School of Medicine and Health Sciences, Monash University Malaysia, Bandar Sunway 47500, Selangor, Malaysia; nurshariza.abdullah1@monash.edu (N.A.); nur.adnan@monash.edu (N.A.A.A.); nur.omarmacha@monash.edu (N.O.M.); 2 School of Pharmacy, Monash University Malaysia, Bandar Sunway 47500, Selangor, Malaysia; nafees.ahemad@monash.edu; 3 Infectious Diseases and Health Cluster, Tropical Medicine and Biology Platform, Monash University Malaysia, Bandar Sunway 47500, Selangor, Malaysia; 4 Institute of Immunology and Immunotherapy, Centre for Liver and Gastrointestinal Research, University of Birmingham, Birmingham B15 2TT, UK; kxa454@alumni.bham.ac.uk; 5 Institute of Biological Sciences, Faculty of Science, University Malaya, Kuala Lumpur 50603, Malaysia; jasmine@um.edu.my; 6 Faculty of Industrial Sciences & Technology, University Malaysia Pahang, Lebuhraya Tun Razak, Gambang, Kuantan 26300, Pahang, Malaysia; adamlee@ump.edu.my; 7 Immunogenetic Unit, Allergy and Immunology Research Center, Institute for Medical Research, Ministry of Health Malaysia, Shah Alam 40170, Selangor, Malaysia; sitiaisyah.aa@moh.gov.my

**Keywords:** alphavirus, nsP3, HVD, indel, repetition, duplication, mutation, evolution, phosphorylation, emergence

## Abstract

Alphavirus non-structural proteins 1–4 (nsP1, nsP2, nsP3, and nsP4) are known to be crucial for alphavirus RNA replication and translation. To date, nsP3 has been demonstrated to mediate many virus–host protein–protein interactions in several fundamental alphavirus mechanisms, particularly during the early stages of replication. However, the molecular pathways and proteins networks underlying these mechanisms remain poorly described. This is due to the low genetic sequence homology of the nsP3 protein among the alphavirus species, especially at its 3′ C-terminal domain, the hypervariable domain (HVD). Moreover, the nsP3 HVD is almost or completely intrinsically disordered and has a poor ability to form secondary structures. Evolution in the nsP3 HVD region allows the alphavirus to adapt to vertebrate and insect hosts. This review focuses on the putative roles and functions of indel, repetition, and duplication events that have occurred in the alphavirus nsP3 HVD, including characterization of the differences and their implications for specificity in the context of virus–host interactions in fundamental alphavirus mechanisms, which have thus directly facilitated the evolution, adaptation, viability, and re-emergence of these viruses.

## 1. Introduction

The *Alphavirus* genus belongs to the Togaviridae family, together with *Rubivirus* and unclassified Togaviridae genera. To date, 32 different *Alphavirus* species have been identified [1], which are globally distributed across all continents except Antarctica. Besides being classified based on their antigenic characteristics, alphaviruses are also categorized as being either New World (NW) or Old World (OW) alphaviruses based on their E1 protein genetic diversity and the geographic locations where they were first isolated [2,3,4,5,6]. 

New World (NW) and Old World (OW) alphaviruses share similarities in a few biological aspects, such as the organization of their genome, their general replication strategies, and the characteristics of their replicase enzyme protein, namely, RNA-dependent RNA polymerase (RdRp) [7]. However, the NW and OW alphaviruses differ in terms of the severity of symptoms they induce and also in terms of many fundamental mechanisms, such as viral replication, pathogenesis, and regulation of host stress responses [8,9]. Interestingly, these differences also exist within the intergroup of NW and OW species, and even among strains of the same species [4,10,11,12]. Many studies have demonstrated that the unique characteristics of alphavirus nsP3 contribute to these functional differences. The alphavirus nsP3 has been demonstrated to be involved in replication and viral transmission, either inter-host or among different hosts. It also plays a role as a determinant of alphavirus virulency and vector specificity, and regulates host stress responses during viral infection. These roles and functions are based on specific virus–host modes of interactions, i.e., between specific individual alphavirus nsP3 HVDs and specific sets of host cellular proteins [13]. It has been proposed that these interactions are employed when alphaviruses adapt to new hosts [3], and that they are also additional significant factors contributing to alphavirus pathogenesis and evolution [14]. 

Alphavirus nsP3 is a ~60 kDa protein with lengths varying from 469 to 570 aa [3,15] that is characterized by three distinct structural domains: the *N*-terminal macrodomain, the alphavirus unique zinc-binding central domain (AUD), and the C-terminal HVD [16,17]. Like other natural proteins, the macrodomain and AUD are globular proteins [16,18]. The alphavirus nsP3 HVD lacks a stable secondary structure or is completely unstructured [2,7,19]. It is a natively unfolded protein and has intrinsically disordered formation in solution [2,3,4,20,21]. Functionally, the nsP3 HVD is a highly flexible region of alphaviruses due to its disordered characteristics and variability in size and aa sequence [2,7,16,22]. These characteristics allow nsP3 HVD to interact with various important cellular host factors at the early stages of viral infection whilst affecting the host cell biology [2,3,23]. 

The present review compiles and summarizes recent genetic research on alphavirus nsP3, specifically its HVD, and discusses potential mechanisms linked to its genetic information. It focuses on the roles and effects of indel, repetitive, and duplication events occurring in the alphavirus nsP3 HVD in determining the specificity of virus–host interactions in fundamental viral mechanisms. These ubiquitous mutation events have contributed to alphavirus evolution, viability, and re-emergence.

## 2. Alphaviruses

The NW alphaviruses include the Venezuelan equine encephalitis virus (VEEV), Western equine encephalitis virus (WEEV), Eastern equine encephalitis virus (EEEV), and Aura virus (AURAV) and are disseminated in the Americas (North America, Central America, South America, and Caribbean Islands) [4,24]. Infections caused by these viruses are likely to cause highly debilitating diseases, often leading to meningoencephalomyelitis and fatal outcomes for the affected host [25]. The OW alphaviruses include the Sindbis virus (SINV), Semliki Forest virus (SFV), Chikungunya virus (CHIKV), O’nyong’nyong virus (ONNV), Ross River virus (RRV), and Barmah Forest virus (BFV), which are mostly distributed across Africa, Asia, Europe, Australia, and Oceania. The Mayaro virus (MAYV), which is also categorized as an OW virus, is currently circulating in South America [4,24,26]. OW viruses are less pathogenic than NW viruses, usually causing milder symptoms such as fever, rash, and arthritis in humans [10,27], and are rarely fatal [28]. Recent outbreaks worldwide have led the OW CHIKV to spread to NW regions [5]. Alphaviruses are pathogenic and can usually cause acute and persistent infections in humans and animals depending on the virus and host type. In invertebrate hosts such as the mosquito, these viruses cause acute and persistent but asymptomatic infections [3,29,30,31,32]. There are currently no licensed vaccines or antivirals available for any alphaviruses, despite the significant threat they pose to worldwide public health due to their epidemic potential [3,11,33,34].

**Figure 1:**
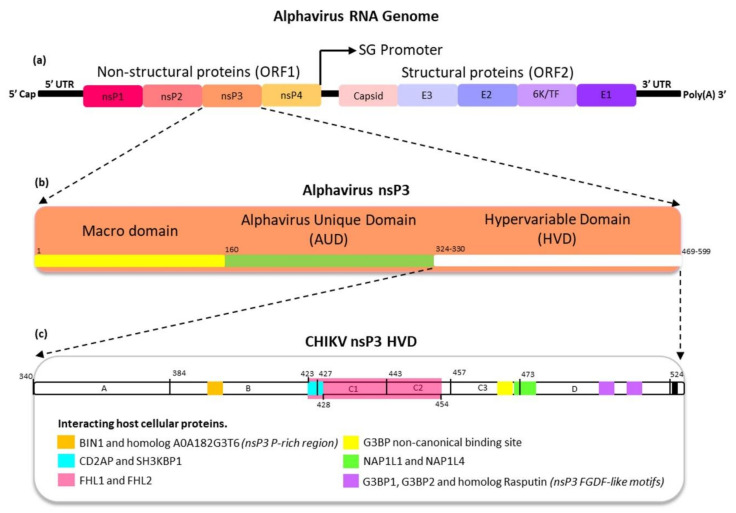
A summary of the alphavirus ss(+)RNA genome and nsP3 characteristics with putative binding regions for various host proteins. (**a**) The ~12 kb alphavirus RNA genome encodes four non-structural proteins (ORF1) and six structural proteins (ORF2), including the newly discovered TF protein. These ORFs are flanked by 5′ and 3′ UTRs. (**b**) The size of the alphavirus nsP3 ORF may vary (1–469/599 bp) and contains three domains: the macrodomain at position 1–160 bp (yellow), the alphavirus unique domain at position 161–324/330 bp (green), and the hypervariable domain at position 324/331–469/599 (white). (**c**) The nsP3 hypervariable domain (HVD) with putative binding regions for various interacting host proteins indicated according to the most recent available information. The putative nsP3 regions interacting with host proteins have been highlighted with different colors. UTR—untranslated region; ORF—open reading frame; SG—subgenomic promoter; Poly(A)—polyadenylation; BIN1—bridging integrator 1; CD2AP—CD2-associated protein; SH3KBP1—SH3 domain containing kinase binding protein 1; FHL1—four and a half LIM domain protein 1; FHL2—four and a half LIM domain protein 2; G3BP—ras-GTPase-activating protein (Ras-GAP) SH3 domain binding protein; G3BP1—ras GTPase-activating protein-binding protein 1; G3BP2—ras GTPase-activating protein-binding protein 2; NAP1L1—nucleosome assembly protein 1 like 1 and NAP1L4—nucleosome assembly protein 1 like 4. This figure is modified from Ahola and Merits, 2016; Gotte, Liu, and McInerney, 2018; Meshram et al., 2018; and Schnierle, 2020.

Alphavirus is a small enveloped virus with a diameter of approximately 65 to 70 nm and a positive-sense single-stranded RNA (ss(+)RNA) genome of approximately 11.5 to 12 kb in size [2,4,23,35]. The alphavirus RNA genome has a 5′ cap and 3′ polyadenylate (poly(A)) tail [36], structurally mimicking the cellular host mRNA [8]. The genome consists of two open reading frames (ORF), flanked by 5′ and 3′ untranslated regions (UTRs) [37], that encode four non-structural proteins (nsP1, nsP2, nsP3, and nsP4 from the 49S RNA genome (G)) and five structural proteins (the capsid (C), E3, E2, 6K, and E1 from the 26S RNA subgenome (SG)) [16,23] (Figure 1a). In 2008, a small ~8 kDa structural accessory protein was discovered, and is known as the transframe protein (TF) [38,39]. The structural and non-structural proteins of all alphaviruses share an amino acid (aa) identity of 45% and 60%, respectively [4]. The nucleotide substitution rate for the alphavirus has been estimated to be between 0.01 × 10^−3^ and 0.24 × 10 ^−3^ substitutions/site/year [40].

### Lifecycle of Alphavirus

Most of our knowledge of the alphavirus replication cycle has been derived from studies of SINV and SFV [16]. The replication and propagation of alphavirus starts with its entry into susceptible and permissive host cells [41], including blood monocyte-derived macrophages [42]; human epithelial, endothelial, and primary fibroblast cells [42,43]; and, in the case of CHIKV, also neurons and glial brain cells [44].

The type of receptor(s) present on the host cell surface for alphavirus E2 glycoprotein attachment and the involved mechanism(s) remain unclear. This is due to the specificity of virus–host interactions, and these aspects remain to be elucidated [16,24,45]. However, there are various ubiquitous receptors that have been suggested, depending on the alphavirus species and the type of host cell, i.e., invertebrate or vertebrate. For example, earlier studies have shown that alphaviruses require membranous proteinaceous receptor(s) for their attachment to the host cells [46,47]. The C-type lectins DC-SIGN and L-SIGN allow SINV to attach to and replicate in primary human dendritic cells as well as human monocytic THP-1 cells that have been transfected to express those lectins [48]. Another study has shown that the level of infection by SINV increased when cell surface laminin was overexpressed in hamster cells, while monoclonal antibodies to human laminin-binding proteins 4F6 and 8E4 have been demonstrated to hinder VEEV replication [49,50]. The natural resistance-associated macrophage protein (NRAMP) mediates SINV binding, facilitating the infection of *Drosophila* cells, while its human homolog, NRAMP2, mediates the binding and entry of the virus into murine cells [51]. Glycosaminoglycan heparin sulfate contributes to increasing the viral infection and virulence of circulating EEEV and SINV with one or more selective mutations in their E2 glycoprotein [52,53,54]. Glycosaminoglycan heparin sulfate has been suggested to act as an attachment factor in enhancing virus–receptor binding [55,56,57]. Additionally, the collagen-binding α1β1 integrin (CD49a/CD29) is responsible for initiating RRV infection in mammalian cells [58]. In addition, heat shock protein (Hsp60), protein prohibitin 1 (PHB1), far upstream element-binding protein 2 (FBP-2), and transferase enzyme phosphoglycerate mutase 1 (PGAM) have been described as CHIKV-binding proteins [59]. Human T-cell immunoglobulin and mucin domain-containing protein (TIM1 and TIM4) and a human family member of Axl have also been indicated to be involved in promoting alphavirus infection [60]. Recent studies have identified Mxra8 as a protein that binds CHIKV, RRV, ONNV, and MAYV [5,61].

After E2 glycoprotein attaches to the receptor(s) on the host cell surface, the alphavirus E1 glycoprotein will then initiate a low-pH-triggered fusion between the virus membrane and the host endosomal membrane [62,63], leading to virus internalization into the host cell via clathrin-dependent endocytosis for CHIKV, SFV, and SINV [43,64,65]. However, a study from 2010 suggests that CHIKV entry is mediated by the Eps15-dependent endocytosis pathway in a manner that is independent of clathrin [66]. Interestingly, another study suggested that a different pathway mechanism might be used by CHIKV during the entry stage, depending on the specific host cell type [59].

Once in the host cytoplasm, the host ribosome will then uncoat the alphavirus RNA genome from its nucleocapsid [23], allowing the alphavirus ss(+)RNA viral genome—containing sequences encoding 42S G (non-structural protein) and 26S SG (structural protein)—to be released [3]. The translation of 42S G by the host ribosome results in the assembly of P123 and P1234 polyproteins [4,23,67]. The P123 and P1234 polyproteins are then transported to the host plasma membrane (PM) and endosomal membranes. The polyproteins bind to these membranes by the specific alpha-helical peptide and palmitoylated aa of nsP1, which acts as an anchor to the membrane [68,69,70].

At the plasma membrane, the nsP2, which has both protease and helicase activity [71], cleaves the P1234 into P123 and P4, separating the individual P4 into RdRp and forming the replication complexes (RCs) containing P123 and P4 [72,73]. These RCs bind to the plasma and endosomal membranes and exhibit enzymatic activities that play important roles in alphavirus RNA synthesis [2,68].

The RdRp P4 later synthesizes the negative-strand RNA using the alphavirus ss(+)RNA viral genome as a template. The newly produced double-stranded RNA (dsRNA) induces the formation of membrane spherules. Membrane spherules have protruding bulb-shaped membrane evaginations at the PM. They consist of RNA complexes connected to the host cytoplasm by a narrow bottleneck structure [23]. The cleavage of P123 and P4 into P1, P23, and P4, and then into P1, P2, P3, and P4 [74], will turn the spherule-associated RCs into a mature form, which is active in synthesizing the G and SG of positive-strand RNA using negative-strand RNA as a template [3,23]. During the early stages of infection, alphavirus spherules are detected at the plasma membrane, while at later stages of infection, they are detected at the endosomal and lysosomal membranes. In the late stages of SFV infection, the spherules are internalized and transported to the perinuclear area, forming cytopathic vacuoles [75].

The 26S SG is translated by free ribosomes, resulting in alphavirus structural polyproteins: capsid–E3–E2–6K–TF–E1. These structural proteins are known to be involved in virion assembly. However, only some of them are incorporated into the virion. The viral capsid, which is the first to be translated in the cytoplasm, uses its autoproteolytic C-terminal domain to cleave itself from the structural polyprotein. The newly synthesized alphavirus ss(+)RNA interacts with the capsid protein via oligomerization to produce the nucleocapsid. Following capsid cleavage, the E3 protein becomes exposed and produces a signal for the remainder of the E3–E2–6K–TF–E1 polyprotein to be translocated across the endoplasmic reticulum (ER) membrane. During alphavirus envelope formation, E2 and E1 form a heterodimer complex with help from the E3 protein. E3 acts as a clamp, holding the E2–E1 dimer together to avoid premature disassembly. It also protects the dimer from exposure to low pH along the host secretory pathway from the ER to the cell membrane. The nucleocapsid then binds to the cytoplasmic tail of E2 to form viral particles and initiate the budding of enveloped virions to the extracellular medium. For most alphaviruses, the 6K protein is suggested to act as an ion channel, and the E3 protein is not incorporated into virions [23,24,76,77]. Though the exact details have yet to be discovered, palmitoylated TF has been demonstrated to be crucial for virus assembly [78].

## 3. The Functions of Alphavirus nsP3

The nsP3 has been proven to mediate many virus–host protein–protein interactions, particularly during replication, in addition to playing a role as a vector specificity determinant and a major viral virulence determinant in the case of some alphaviruses [3]. Previous studies have also described nsP3 as a regulator of host stress responses and also as a transmission agent, both inter-host and between hosts. Interestingly, the localization of alphavirus nsP3 in infected host cells depends heavily on its putative roles and functions while interacting with various host proteins during the formation of viral RC and nsP3-containing cytoplasmic granule complexes [79].

### 3.1. Replication

The nsP3 is known to be essential for the viral RNA synthesis [2,80] even though it has been suggested to have indirect contributions to the mechanism, and its actual role in this context remains under investigation [2,14].

There is an increasing amount of data showing that both NW and OW alphavirus nsP3 has been demonstrated to interact with various host proteins, and its HVDs function as hubs where viral and cellular host proteins gather. This assembly is part of the early alphavirus RC construction mechanism, forming the nsP3-RC, which has been demonstrated to mediate viral G and SG RNA replication [9,80,81,82,83,84,85,86,87,88].

Interestingly, nsP3 HVD also contributes to the replication process by mediating the association of replication complexes with the host membrane via its weak peripheral affinity for membranes [89].

### 3.2. Vector Specificity Determinants

Several studies have shown that nsP3 allows NW and OW alphaviruses to adapt their replication processes to different mosquito vector species. To date, distribution of each alphavirus has been limited by the particular ecological conditions of their specific host reservoir vector [90,91]. For example, VEEV uses the *Culex* (*Melanoconion*) as its primary vector [92]. This mosquito subgenus is distributed only throughout the Americas [93]. Thus, the circulation of VEEV is restricted to Central, North, and South America as a result of this highly specific virus–vector interaction [6].

In 2013, another study demonstrated that construction of a chimeric virus in which the CHIKV backbone was fused to the gene for the entire ONNV nsP3 resulted in it becoming infectious, increasing its replication rate from 0% to 63.5% in *Anopheles gambiae* [94]. This was an important discovery as CHIKV and other alphaviruses do not normally infect and replicate in *An. gambiae*. However, ONNV is an exception, as it is transmitted by *Anopheles* mosquitoes [95]. Interestingly, despite the differences in transmission vector types, the nsP3 of CHIKV and ONNV share 81% and 72% similarity at the amino acid and nucleotide level, respectively. The study also showed that a chimeric with a CHIKV backbone and ONNV nsP3-C-terminal-containing HVD can increase the infection rate in *An. gambiae* to 9.1–17.8% [94,96]. This finding suggests that nsP3, particularly the C-terminal with the HVD region, is responsible for specific molecular interactions with the host cellular factors. Additionally, this demonstrates that nsP3 is important in determining the specificity of possible vector species for the virus [96].

As suggested by previous studies, CHIKV nsP3 HVD may play a role in determining infection in specific mosquitoes. A study in 2018 showed that the interactions between two conserved motifs in CHIKV nsP3 HVD—the proline-rich (P-rich) region and the FGDF-like duplicate motifs—and *Aedes aegypti* cellular proteins are important in the infection and dissemination of CHIKV in the *Ae. aegypti* mosquito vector [96].

These examples indicate that there is a distinct relationship between alphavirus nsP3—particularly HVD and its conserved motifs—and mosquito vector proteins, which determine the specificity of alphavirus infections and virulency.

### 3.3. Viral Virulence Determinants

Virulence is the ability of a virus to cause disease or act as a pathogen. There are four categories of viral protein functions that are responsible for virulence: (i) proteins that affect virus replication ability, (ii) proteins modulating the host defense mechanism, (iii) proteins assisting virus dissemination, and iv) proteins which are toxic toward the host. Many proteins fall into more than one category [97].

In addition to alphavirus proteins E2 [98,99,100,101,102,103], capsid [104,105,106], nsP2 [71,107,108,109,110], and E1 [111,112], as well as the 5′ untranslated region [102,113,114,115], alphavirus nsP3 is also a major virulence determinant [15,95] for OW and NW alphaviruses. These alphaviruses include SINV [112], SFV [22,116,117], CHIKV [2], and EEEV [14]. A study in mice showed that the nsP3 protein also shows strong potential to perform the same function for VEEV, depending on the mouse’s age and VEEV genotype [118].

Studies have shown that the nsP3 protein can influence neurovirulence in mice. An avirulent A7(74) SFV strain became a lethal neurovirulent when its nsP3 was replaced with nsP3 from virulent SFV strains [22,116,117]. The modified A7(74) also became more virulent when it expressed additional nsP3 proteins from virulent strains SFV4 and SFV6. Based on these findings, researchers have focused on two nsP3 domains, the nsP3 macro and HVD, which are possibly responsible for forming alphavirus-virulent elements.

Two nsP3 macrodomain functions—namely, the binding and hydrolysis of ADP-ribose (ADPr) from ADP-ribosylated proteins—have been suggested to play important role(s) in OW alphavirus, including SINV and CHIKV replication and virulence in vitro and in vivo. Studies have revealed that impairing both binding and hydrolyzing capabilities significantly reduced infection initiation and replication for both SINV and CHIKV [119,120]. The binding of ADPr is crucial for the initiation of viral replication, while mono-ADP-ribosyl (MAR) hydrolase helps in viral RC amplification. However, their molecular pathways and proteins networks are still poorly understood [15,119,120,121,122,123].

Interestingly, besides the macrodomain, nsP3 C-terminal HVD has also been determined to play a role in alphavirus SFV, SINV, and EEEV neurovirulence [124]. In SFV, the mutations that cause nsP3 phosphorylation activities to be defective lower the viral RNA synthesis rate and significantly reduce SFV virulence in mice [89]. Meanwhile, the deletions in its C-terminal HVD inhibit SG RNA production and infection establishment, change nsP3 cellular localization, and also reduce virulence in mice [13,124].

For EEEV, two HVD conserved motifs—namely, the fragile X syndrome protein (FXR)-binding motif and RasGAP SH3-binding protein (G3BP)-binding sites—are responsible for its virulence. The deletions of these sites make EEEV no longer neurovirulent for mice and affect its viral replication efficiency, both in vivo and in vitro [14,87]. The existence of a single mutation event in E1 and E2 and two mutations in nsP3, including a natural 18 aa deletion in the C-terminal [101], is required for SINV virulence [112].

The existence of an opal stop in the nsP3 HVD region has been suggested to contribute to alphavirus virulence. For ONNV, evolution pressures have allowed its population to maintain both nsP3 ORF sequences, with each consisting of either an arginine codon or an opal stop codon, which produce polyproteins P1234 and P123, respectively. The maintenance of both sequences indicates that they are both needed for ONNV’s natural life cycle [95,125,126]. The exact role of the opal stop codon in alphavirus virulence is still unclear. However, previous studies have demonstrated that the existence of the opal stop codon (i) provides ONNV with the capability to infect mosquitoes [125], (ii) plays a role in SFV pathogenesis [116,127], and (iii) is associated with virulence increment in SFV [22] and SINV, as one of three important nsP3 mutations for AR86 strains [112]. It also plays a role in inducing severe CHIKV pathogenesis [128].

From these extensive studies, we may conclude that the (i) nsP3 macro–ADP, (ii) nsP3 HVD phosphorylation sites, (iii) HVD–FXR binding site, (iv) HVD–G3BP binding sites, and (v) opal stop codon are important for alphavirus virulency. However, we must acknowledge that there are other structural and non-structural viral proteins, a complete set of nsP3-interacting proteins, cis-acting elements of the alphavirus genome, and other host factors involved in the orchestration of fundamental virus functions, which are multi-component events [14,95,117]. Most importantly, alphavirus virulent mechanisms are highly dependent on specific alphavirus species and the concentrations of essential host factors in specific cell lines [118].

### 3.4. Regulation of Host Stress Responses

The ability of nsP3 to interact with various cellular proteins and functioning as gathering hubs also has allowed the formation of large cytoplasmic complexes [9,10,14,87,129,130,131], which were suggested to regulate the host stress response during viral infection [108,129]. The OW alphaviruses SFV, SINV, CHIKV, and RRV and the NW alphavirus EEEV interact with mammalian G3BP family members and its mosquito homolog, Rasputin (Rin), forming the HVD–G3BP and HVD–Rin granule complexes in the cytoplasm of mammalian and mosquito hosts, respectively [32,132,133]. During OW alphavirus infection, nsP3 granules in the cytoplasm sequestrate the diffuse cytoplasmic G3BP or Rin into a granular form using its AUD and HVD, subsequently forming HVD–G3BP or HVD–Rin cytoplasmic granules [10]. Two conserved FGDF-like motif(s) and unidentified regions in nsP3 HVD are involved in the G3BP- or Rin-binding interactions and sequestration processes [10,129]. The NW alphaviruses VEEV, EEEV, and WEEV’s nsP3 HVD sequestrate one of the FXR family, the Fragile X mental retardation protein (FMRP)—which is involved in stress granule formation. The FMRP was suggested to suppress viral RNA translation by redirecting it into the host’s stress granules. The interaction site is known as the Agenet-like domain binding motif [9,87,131,134]. Subsequently, the alphavirus nsP3 HVD is suggested to modulate the host stress response by interacting with stress granules’ components and preventing host stress granule formation [32,131,132,133].

### 3.5. Transmission Agent for Inter-Host and among Hosts

Studies of the phylogenetics and evolution within the ORF of the RRV genome have led to the identification of between 106 and 830 individual sites that have undergone highly significant negative selection pressure and 5 sites that have undergone positive selection pressure since the start of its divergence from its ancestral strain 94 years ago. Interestingly, one of the 5 positive selection pressure sites is at the 333 aa position of nsP3 HVD [135,136]. These findings are in agreement with a previously suggested theory that low-fidelity mosquito-borne RNA viruses, such as alphaviruses, have limited evolution due to the difference in replication mechanisms between mosquito and mammalian host systems [40,137,138]. Genetic evolutions that increase the virus fitness in a mammalian host might cause replication inhibition in mosquito vector hosts, and vice versa [138]. Thus, this phenomenon, which involves RRV nsP3 HVD at the 333 aa position as a positive selection pressure site, does clearly affect the rate of alphavirus transmission among its hosts.

In addition, a previous study demonstrated the importance of the interaction between CHIKV nsP3 HVD and Rasputin, an *Aedes albopictus* cellular protein, for viral pathogenesis [108]. When the in vivo interaction was absent, the CHIKV infection rate and transmission in live *Ae. albopictus*, from its body (abdomen and thorax) to its head (saliva), significantly decreased.

All other alphavirus nsPs, including nsP1, nsP2, and nsP4, are also known to play important roles in alphavirus RNA replication and translation. As with nsP3, these nsPs have shown complex cellular localization patterns specific for each protein based on their functions. For example, they assemble in RCs and are present in various locations, depending on their individual virus–host interactions [16]. The nsP1 acts as an anchor for viral replication complexes at the host membrane, possibly interacting with anionic phospholipids in the host membrane [139,140,141,142]. Together with nsP4, it is also involved in the synthesis of negative-strand RNA [80,143]. In addition, it plays a role in the methylating and capping of positive-strand mRNA, preparing it for translation while also protecting it from cellular 5′ exonuclease activity [21,144,145,146]. nsP2 has several enzymatic activities, such as proteinase and helicase activity, which have specific functional roles. The proteinase function of nsP2 allows for non-structural polyprotein processing necessary for viral translation and replication. The *N*-terminal RNA helicase activity of nsP2 has seven superfamily 1 (SF1) helicase signature motifs, with both nucleotide triphosphatase (NTPase) and RNA triphosphatase activities [21,67,147,148,149,150]. The helicase is used in viral RNA genome replication and viral SG transcription [151,152]. Thus, nsP2 acts as an alphavirus virulence factor and interferes with the host cellular antiviral responses via several mechanisms. For example, it can shut off transcription and translation in the infected host. Furthermore, it can inhibit the host antiviral infection response, such as the interferon-induced JAK–STAT signaling pathway, allowing viral factors to control the host translational machinery. Finally, nsP2 can inhibit the unfolded protein response (UPR) cellular defense mechanism. During viral infections, viral proteins are translocated into the ER for post-translational modifications and proper protein folding. The increased concentration of viral unfolded and misfolded proteins in the ER induces the UPR mechanism. Upon activation, UPR increases the production of proteins responsible for protein folding, degradation, and apoptosis, thus promoting cell survival [71,107,108,109]. The nsP4 acts as the RdRp, having an RdRp domain at the C-terminal end. This protein is responsible for synthesizing the negative-strand RNA genome using the ss(+)RNA genome virus as a template in preparation for producing dsRNA for mature RC–membrane spherule construction. It is also responsible for replicating the viral RNA genome using negative-strand RNA as a template to produce new copies of the ss(+)RNA alphavirus genome. In addition, nsP4 is involved in SG RNA transcription toward production of structural proteins for virion production [4,153].

## 4. The Protein Domains of Alphavirus nsP3

Alphavirus nsP3 has three domains: the macrodomain, the alphavirus unique domain (AUD), and the hypervariable domain (HVD). To date, only the crystal structures of the macrodomain for CHIKV and VEEV [17,21], parts of the nsP2 protease and methyltransferase-like domains and the nsP3 macro and AUD zinc-binding domains for SINV have been resolved [21,154]. The NMR structures of the nsP3 macrodomains of CHIKV, VEEV, and MAYV also have been previously solved [155,156,157]. Remarkably, a recent study using a novel NMR strategy revealed that even though the VEEV nsP3 HVD is dominantly disordered, some fragments do form secondary structures. This includes a short stable α-helix structure, which has been previously suggested to interact with the FXR family [19]. Based on crystal structures, the macrodomain of CHIKV and VEEV reveals that its structure is very similar to that of the homologous domain in *Escherichia coli* [17].

### 4.1. Macrodomain

The ~150–160 aa macrodomain is located at the *N*-terminal of alphavirus nsP3 [16]. It is highly conserved among alphaviruses and other positive-strand RNA viruses, such as coronaviruses, rubella, hepatitis E viruses, and unrelated coronaviruses [2,16,158,159,160,161]. The macrodomain is a six-stranded, twisted, centrally located β-sheet surrounded by three α-helices on one side and one on the other side. The combined structures of the four α-helices and six β-strands are preserved in all OW and NW alphaviruses [15]. A narrow opening within this region interacts with mono-adenosine diphosphate-ribose (ADPr) and poly-adenosine diphosphate-ribose (PAR) through mono- or poly-ADP-ribosylation, which is a post-translational modification process. It is also bound to negative-charge polymers such as RNA [17,130,162,163]. The macrodomain narrow opening is a potential target for antiviral development, as inhibitors could fit into the crevice-binding pocket [164,165,166]. The SFV nsP3 macrodomain has a putative recognition site for the nsP2 protease to cleave the polyprotein P23 during viral replication [167]. The macrodomain has also been determined to have a detectable hydrolase activity level by hydrolyzing the ADP-ribose groups from mono(ADP-ribosyl)ated proteins. This activity might be crucial for CHIKV replication and virulence in vivo [17,119,168,169]. Moreover, the macrodomain is involved in alphavirus G RNA replication by gaining adaptive mutations due to modifications in alphavirus RNA promoter elements [2,170,171]. The residue M129 is involved in interactions with a mosquito RNAi component which inhibits the vital antiviral pathway for mosquitoes [172]. The nsP3 macrodomain is also suggested to affect the phosphorylation of nsP3, P23 processing, the synthesis of negative-strand RNA, and the inhibition of host protein synthesis [119,121,122]. However, the mode of action is still poorly understood [173].

### 4.2. Alphavirus Unique Domain (AUD)

AUD is a small, intermediate linker domain [130], also known as a central zinc-binding domain [3]. It is located at the center of the nsP3 genome structure, between the nsP3 macro domain and HVD. AUD is conserved only among alphaviruses with a high level of sequence homology, and no homolog is present in the non-structural proteins of other viruses [4,127,154,172,174]. This domain is formed by two parallel β-sheets and antiparallel α-helices consisting of many serines and threonines. Four of its conserved cysteines, all of which are essential for alphavirus replication, bind to a structural zinc ion (Zn^2+^) and form a putative binding surface for RNA [3,154,172]. Though its functions are still under investigation, the high conservation of the alphavirus AUD sequence indicates that it is essential to the alphavirus life cycle [172]. This domain also has pleiotropic functions for the life cycle of alphaviruses. It is involved in P23 polyprotein cleaving, the formation of RC during negative-strand RNA synthesis, virion assembly, and also alphavirus infectivity [16,81,82,127,154,172]. It has also been shown that adaptive mutations in AUD could counterbalance changes in nsP3 HVD [171,175]. Interestingly, a recent CHIKV AUD mutagenic analysis has shown that residues R243 and K245 together, and P247A/V248A of AUD are critical determinants of virus–host specificity. R243 and K245 are required for CHIKV genome replication, while P247A/V248A are involved in CHIKV entry and release, but not the assembly of infectious viral particles.

Several important characteristics of AUD function can now be distinguished, such as (i) acting as a counterbalancing functional unit for nsP3 HVD change events; (ii) acting as a virus–host specificity determinant in viral replication mechanisms; and (iii) possible involvement in interference of the host cellular RNAi antiviral response. This small domain also offers putative targets for antiviral and attenuation vaccine development [172]. However, to date, there remains much to be discovered regarding the roles of AUD in the viral and host protein network and their effects on the alphavirus phenotype.

### 4.3. Hypervariable Domain

HVD is also known as the C-terminal or the ‘tail’ region of alphavirus nsP3. This region starts at position 324–330 of nsP3 [2] and can tolerate large insertions and deletions [12], allowing its length to be highly variable, though it is typically over 200 aa [2,19]. The nucleotide and amino acid sequences of HVD are non-conserved, even between closely related alphaviruses from the same serocomplex [2,3,13,14,16,162] and among strains of the same alphavirus species [3]. The HVD is intrinsically disordered and has little tendency to form secondary structures [19]. Interestingly, nsP3 is the only protein which can be phosphorylated among all alphavirus replicase proteins. The nsP3 HVD is heavily phosphorylated and is involved in signaling cascades, alphavirus RC formation, and alphavirus virulent phenotypes [176,177]. Structural studies on nsP3 HVD are key to gaining insight into alphavirus RC assembly and functions during viral replication at the molecular level.

## 5. The Functions of Alphavirus nsP3 HVD

The nsP3 HVD functions as a hub allowing interaction of various host proteins [177]. Despite being highly variable and having non-conserved genetic characteristics, nsP3 HVD from different species of alphavirus still consist of several conserved identified linear motifs that have been proven to interact with specific sets of host cellular factors from at least three protein families. These include several protein families with SH3 domains, either the G3BP, FXR, or both family proteins, and also two members of the NAP1 family [2,9,10,12,20,79,86,87,131]. Interestingly, particular HVD linear motifs are short, repetitive, and located at the very end of the HVD C-terminal [9,129] and demonstrate a high redundancy in functions during RC assembly and alphavirus replication [2,87].

Many reviews and scientific papers have discussed nsP3 characteristics and the specific protein–protein interactions between nsP3 HVD and host cellular proteins. Examples include the following: (i) the alphavirus molecular mechanism on viral RNA replication and interactions with its host cells [178,179]; (ii) the characteristics and functions of alphavirus nsP3 [3,14,15,118]; (iii) the protein–protein interaction of alphavirus CHIKV nsPs with its host factors as antiviral targets [180]; (iv) the specific virus–host protein interactions of OW and NW alphavirus nsP3 with their hosts [2,8,9,87,162,171]; and (v) the host cellular proteins which interact with alphavirus nsP3 [2,10,12,15,129,181,182,183,184].

### 5.1. Alphavirus Proline-Rich Region Interactions with SH3-Domain of Host Cellular Proteins

The alphaviruses SFV, SINV, and CHIKV conserved the P-rich region, which has a Src homology-3 (SH3)-binding domain, binds to the C-terminal SH3 domain of bridging integrator-1 (BIN1), also named amphiphysin-2 or SHSP9 protein, and its mosquito homolog A0A182G3T6 [2,9,79,96,185]. One study suggested a new extended P-rich SH3-binding motif, P*XX*P*X*Rp*X*R, for SFV, emphasizing two constant arginine residues which will provide additional positive charges, leading to an extremely high affinity for binding the SH3 domain [185]. However, only SFV has the extended version of the P-rich region as mentioned, while SINV and CHIKV have a shorter initial motif, P(I/V)(P/A)PPR [3]. The number of P-rich regions of the nsP3 HVD and their positions can vary according to the alphavirus species, for example, there is one region in MAYV, SFV, CHIKV, ONNV, and SINV; two regions in NW alphaviruses VEEV, EEEV, and WEEV; and four regions in RRV and BFV—all found at different positions within nsP3 HVD [2,37,79,136,186].

One study determined the CHIKV nsP3–SH3 domain BIN1 solution-state structure using NMR spectroscopy [185]. Previous studies have suggested that Bin1 plays prominent roles in cellular mechanisms, such as in regulating endocytosis and membrane recycling, cytoskeleton regulation, DNA repair, cell cycle progression, and apoptosis [185,187]. The nsP3 HVD P-rich region has been suggested to recruit BIN1 to viral RC [79] and also to recognize and induce membrane curvature [16,188], though the latter has yet to be confirmed [3]. The nsP3 HVD P-rich–SH3 domain interactions are critical for alphavirus replication in mosquito cells rather than in mammalian cells [2]. Interestingly, in addition to the BIND1 protein, the VEEV nsP3 P-rich region has been identified to bind to the SH3 domain of CD2AP/SH3KBP1, CIN85, and SASH1 proteins [9].

### 5.2. Alphavirus FGDF-Like Motif Interactions with Host Cellular G3BP and Rasputin

The FGDF-like motif is a short peptide motif that is usually in duplicate form at two sites of nsP3 HVD [3,9,129]. These motifs are not conserved in all alphavirus species but are very common among alphaviruses, with one site for MAYV and EEEV and two duplicate sites for the OW alphaviruses, SINV, SFV, CHIKV, ONNV, RRV, and BFV. However, this site does not exist in the other two NW alphavirus species—namely, VEEV or WEEV [3]. These motifs have been demonstrated to bind to the nuclear transport factor 2 (NTF2)-like domain of G3BP family protein [83,129] in vertebrates, and also to the G3BP homolog in mosquito Rasputin [10]. nsP3 HVD FGDF–G3BP binding interactions have been widely observed in SINV, RRV, SFV, and particularly CHIKV infections, while Rin interacts with CHIKV FGDF-like motifs in live *Ae. albopictus* mosquitoes [2,3,9,10,14,85,86,96,129,189]. Both nsP3 FGDF–G3BP and FGDF–Rin interactions provide proviral functions as they are essential for CHIKV replication in different hosts, along with other virus and cellular factors [2]. Deleting one of the FGDF-like motifs does reduce the CHIKV replication level, while the mutation and deletion of both motifs causes a complete loss of FGDF-Rin/G3BP co-localization and inhibits CHIKV propagation in both mammalian and mosquito cells [2,10,96]. Previous studies have suggested that the interactions facilitate replication, starting from the translation of nsPs polyprotein to viral RNA replication [9,181,190]. In addition, G3BP1 and G3BP2 are components of cellular host stress granules [3,16]. Thus, via FGDF–G3BP interaction, nsP3–G3BP cytoplasmic granules are formed and are assumed to indirectly prevent true stress granule formation during CHIKV infection [10,129,133]. Studies have demonstrated that FGDF–Rin interaction is one of the most important alphavirus transmission determinants [10,94,96] since the depletion of Rin severely decreases the CHIKV titer and transmission level in *Ae. albopictus* [10]. Interestingly, besides FGDF-like motifs, it has been suggested that there is another or several other binding site regions in nsP3 HVD for G3BP binding. However, the interaction is low, indicating that the interaction occurs at a low efficiency [2].

### 5.3. Alphavirus nsP3 HVD Interactions with Other Host Cellular Proteins

In addition to the P-rich region and FGDF-like motifs, there are other regions in alphavirus nsP3 HVD that are responsible for alphavirus replication via the virus–host specific mode [2]. These regions are responsible for interacting and binding to a few host cellular proteins, such as members of the FXR and NAP1 families and the FHL1 and FHL2 proteins. The FXR family protein-binding region is present in the nsP3 HVD of NW alphaviruses, including VEEV, WEEV, and EEEV [3,14]. Interestingly, despite having an FXR protein-binding region in their nsP3 HVD, neither VEEV nor WEEV have FGDF-like motifs to bind to G3BP proteins. However, EEEV has both FXR- and G3BP-binding regions, allowing it to interact with proteins of both families. This demonstrates that the OW alphaviruses exclusively interact with G3BP protein, while the NW alphaviruses only interact with the FXR protein, and that EEEV interacts with both G3BP and FXR proteins [3,9]. Like G3BP, the FXR family is also a stress granule-related protein [9]. Thus, both proteins are likely to have the same critical functions during alphavirus replication [152], based on the RNA-binding domain function and regulation of host stress responses [3]. A previous study indicated that both FXR and G3BP have redundant functions during EEEV infection [191], which may be the basis of EEEV’s efficient replication and high level of virulence [14,87]. The relation between OW alphaviruses with G3BP and NW alphaviruses with FXR protein in terms of their similar roles and functions is proof that alphaviruses recruit distinct sets of host proteins that participate in their fundamental mechanisms [3].

NAP1L1 and NAP1L4 are members of the NAP1 family and have been demonstrated to have redundant functions during CHIKV replication via interactions with CHIKV nsP3 HVD [2]. Studies have suggested that the HVD–NAP1 interaction is the primary candidate promoting CHIKV replication in vertebrates, as demonstrated in human Huh 7 and HEK293, mouse NIH 3T3, and hamster BHK21. Interestingly, the NAP1 family protein does not interact with other alphavirus nsP3 HVDs [2,9,87]. Previous studies have demonstrated that the CHIKV nsP3 HVD interacts with FHL1 and FHL2 in human Huh7 and mouse NIH 3T3. However, no interaction between HVD and FHL1 has been found in humans based on studies in HEK293 cells [2,87]. A recent study has also confirmed the findings on FHL1, pointing out that FHL1 is crucial for CHIKV RNA replication. Additionally, the HVD–FHL1 interaction could cause cellular dysfunctions, leading to pain in the muscles and joints of infected vertebrate hosts. This suggests that FHL1 protein is vital for CHIKV infection and pathogenesis in human and mouse hosts [182]. Interestingly, a recent study demonstrated that the FHL1 is not crucial for CHIKV replication; however, its absence did result in slower spread of CHIKV. The study also demonstrated that the CHIKV FHL1- and CD2AP-binding sites overlap (Figure 1c) [192].

### 5.4. Opal Stop Codon

In the alphavirus nsp3 HVD region, an opal stop codon exists near the end of the C-terminal of the nsP3 gene [24], before the cleavage of read-through nsPs polyprotein and 6 aa residues toward the nsP4 protein [16]. In nature, most alphaviruses have a condition in which a leaky stop codon could occur at a low frequency (5–10%) [4,24,29,193,194]. As a result, read-through translation occurs with subsequent low-efficiency expression of P1234 polyprotein and extremely high amounts of P123 polyprotein, allowing the polyprotein P1234 and P123 to co-exist in the same infected host cell [24]. The polyproteins P123 and P1234 of CHIKV in mammalian cells are produced at the early stage of infection, and their production is stopped upon general inhibition of host cellular translation, which usually happens 6–8 h after viral infection [16]. The read-through process is needed for nsP4 RdRp production as part of viral RCs for viral RNA synthesis and transcription processes [152,195]. nsP4 is the first mature virus protein produced during SFV infection [196,197,198]. In the leaky stop codon read-through condition, the nsP4 is expressed less often than other nsPs, suggesting that it is degraded by the proteasome only when it is erratically expressed [67,196,198,199,200].

In other conditions, the replacement of the opal stop codon with other sense codons such as arginine, cysteine, or tryptophan has been observed in several alphaviruses. As a result, a read-through process occurs, and polyprotein P1234 is exclusively expressed in the infected host cell cytoplasm [16,194,201]. For CHIKV, both virus population variants—with the opal stop codon or with the replacement of the opal stop codon with other sense codons, particularly arginine, in nsP3 HVD—exist in nature. However, the majority of strains contain the opal stop codon [3,128,202,203,204]. Due to the co-existence of both virus population variants in nature, with or without the nsP3 stop codon in their genome, it has been suggested that the existence or absence of the nsP3 opal stop codon has little impact on CHIKV [16]. However, it was shown by Jones et al. (2017), in an in vitro and in vivo study involving several vertebrate and invertebrate host cells and specific tissues, that the opal stop codon is crucial in regulating CHIKV replication and promoting CHIKV-induced inflammatory pathology. Interestingly, the pathogenicity induction event was independent and not associated with the CHIKV replication mechanism. However, more studies need to be conducted to determine the mechanism(s) behind their observation [128].

For ONNV, substitution from the opal stop codon to arginine has been observed by the fifth passage in the Vero cell line [194]. Moreover, we also observed the substitution of an opal stop codon for tryptophan in strain HB67652 (accession number MF409176), a human serum isolate from Begoa, Nigeria, in 1966 [126,205]. Studies have suggested that both ONNV population variants, which consist of both the arginine and opal stop codon variant of nsP3 co-existing in nature, provide a quasi-species condition for ONNV. This condition is essential to endure repeated bottleneck events during virus transmission, both within the host and between vertebrate and invertebrate hosts [3,125]. Maintaining this condition of co-existence under evolutionary pressure is very important for ONNV and CHIKV and allows them to sustain different capabilities for alternating their replication and surviving in different hosts [3,125]. Studies have suggested that the opal stop codon is necessary for the establishment of persistent ONNV infection in mosquitoes. Therefore, the establishment occurs through the inhibition of nsP4 RdRp expression, limiting virion replication [95,125].

For SFV, the avirulent strain rA774 possessing the nsP3 opal stop codon was observed to become highly neurovirulent and lethal for adult BALB/c mice when it switched with the nsP3 arginine from a virulent strain, SVF4 [22]. The switch also resulted in limb paralysis for several mice. Interestingly, these two nsP3 variants differ from each other not only in terms of the opal stop and arginine sense codon but also in the deletion of 7 aa in the HVD of rA774. Thus, this study proposes that nsP3 opal stop codon is a vital virulence determinant for SFV, alongside other factors from the entire nsP3 gene [22].

In SINV, substitution from the opal stop codon to cysteine was observed in strain S.A.AR86 [112,206]. A similar replacement in SFV, increased viral virulence in mice [22,112,116,206]. The replacement led to a reduction in both viral G and SG RNA synthesis, an increase in the nsP3/4 polyprotein precursor level, and a reduction in the mature nsP3 level during the early stage of SINV infection [207]. Therefore, more research is needed to determine the mechanism(s) through which cysteine could affect SINV virulence in mice, such as by either modifying non-structural polyprotein processing, viral synthesis, or both, or via an unknown mechanism [112].

In RRV, virus strains K3011 from the G2 lineage and P42213 from the G3 lineage have arginine and cysteine to replace the opal stop codon, respectively. K3011 is a mosquito isolate, and the first case of opal replacement with arginine for RRV was reported in 1990. The isolation of P42212 occurred during the end of the Pacific Island Countries and Territories (PICTs) epidemic in 1980. Interestingly, the transition of the opal stop codon to cysteine is similar to the substitution in the SINV S.A.AR86 strain, which is related to neurovirulence in mice [112,136,206]. However, the exact consequences of opal stop codon substitution in RRV remain an enigma.

In addition, a recent study on VEEV proposed that nsP3 functions as an accumulator to purify viruses with unfit genomes in mutant swarms by targeting them for abolishment. This function occurs during repeated bottleneck events in RNA arbovirus transmission cycles, whether within the host itself or between invertebrate and mammalian hosts [208].

The replacement of the opal stop codon with other sense codons, that allow read-through, has been observed to lower viral transmission in mosquito vectors and significantly reduce the viral pathogenicity of CHIKV, SINV, and ONNV in infected hosts [125,128,207]. In conclusion, the specificity of virus–host interactions is affected by the replacement of the opal stop codon with other sense codons.

## 6. Indel, Repetition and Duplication Events of Alphavirus nsP3 HVD

The nsP3 HVD can endure large aa deletions and insertions [3,16,127]. Here, we discuss a few selected mutation events observed in the nsP3 HVD of different alphavirus species for a better understanding of the characteristics and functions of these events. Alongside the description of these mutation events, we present the multiple sequences alignment (MSA) results for the studied alphaviruses. The motifs mentioned here are based on our own MSA analysis using various alphavirus strains selected from multiple studies. Thus, the motifs and aa positions might be slightly different from the motifs that we have discussed previously. However, the motifs here maintain the overall motif specificity characteristics and are heavily based on previous studies.

### 6.1. Indel, Repetition, and Duplication Events in CHIKV nsP3

CHIKV was first isolated in Tanzania in 1952 [209], and since then it has been responsible for causing multiple sporadic and geographically restricted epidemics in Africa and Asia from 1960 to 1999. The first documented Asian CHIKV outbreak took place in 1958 in Bangkok, Thailand, followed by outbreaks in Cambodia, Vietnam, Laos, Myanmar, Malaysia, the Philippines, and Indonesia [90,210,211,212,213]. CHIKV started to attract the attention of researchers worldwide in 2005–2006, when it re-emerged and caused an explosive outbreak in Indian Ocean islands; approximately 244,000 cases were recorded on Réunion Island by April 2006 [211]. *Ae. aegypti* was the main vector responsible for the human-to-human transmission of CHIKV. However, due to the successful adaptation through E1 glycoprotein aa substitution A226V, CHIKV could replicate more efficiently in *Ae. albopictus* [211,214,215]. Due to this microevolution, CHIKV has expanded its epidemic territory through the urban transmission cycle to urban Asian regions where *Ae. albopictus* is disseminated, such as India, Sri Lanka, Malaysia, Indonesia, Thailand, and Singapore. During the same period, CHIKV E1A226V infected approximately 1.3 million people in India [213,216,217,218,219,220,221,222,223,224]. The CHIKV E1A226V variant has also been disseminated in Europe, including in Italy (2007) and France [210,225], as well as Papua New Guinea (2012) [226]. Later in December 2013, a major CHIKV epidemic occurred in the Caribbean islands [227,228,229] and, subsequently, there were 22,796 confirmed cases out of approximately 1 million suspected cases reported in 2014 in the Americas [226,230].

CHIKV lineages are classified based on the region in which the cases were reported. It has three major lineages: the West African lineage (WA); the East, Central, and South African lineage (ECSA); and the Asian lineage. Two sublineages, the Indian Ocean lineage (IOL) and Asian/American, emerged from the ECSA and Asian lineages, respectively [231]. According to distinct geographical territories and temporal factors, the CHIKV Asian lineage is divided into two clades: the Indian and the Southeast Asian (SEA) clades [225]. Differences in the CHIKV genetic properties among lineages and sublineages have caused differences in epidemiology, pathology, and virulence among CHIKV strains [202,231]. Previous geographically restricted epidemics in Asia were caused by the Asian lineage [213]. The ECSA lineage and IOL A226V substitution sublineage were responsible for outbreaks in 2005 and onwards, such as those in Réunion Island [211,232], Asia, the Indian Ocean islands, and Europe [211,217,233,234]. Meanwhile, the Asian/American sublineage was the major cause of several epidemics in the Pacific Islands and the Americas [225,235].

**Figure 2:**
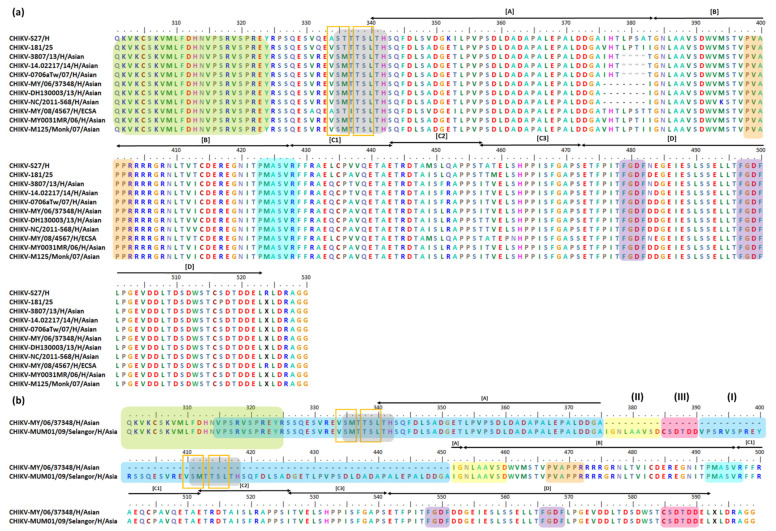
Important motifs and mutation events in CHIKV nsP3 HVD. The HVD starts at position 325. It has one P-rich region at positions (i) 388–393 (**a**) or (ii) 467–472 (**b**) (peach) and two FGDF-like motifs at positions (i) 479–482 and 497–500 (**a**) or (ii) 548–551 and 566–569 (**b**) (purple). For the fragment A of certain CHIKV strains, there is a 4 aa deletion at position 379–382 and a 7 aa deletion at position 376–382 (**a**). For CHIKV MUM001–2009-Selangor, there is a 76 aa duplication event at position 376–456 (**b**). The 76 aa duplication motif is indicated as motif (I) at position 391–456 (blue), motif (II) at position 376–384 (yellow), and motif (III) at position 385–390 (red). The template positions as follows: motif (I) 315–375; motif (II) 452–460; and motif (III) 585–590 (**b**). The PMASVR motif is at position 423–428 (**a**) or 491–497 (**b**) (turquoise), while the *Ae. aegypti* S(M/T)(T/I)TSLTH motif is at position 335–342 (**a**), and its duplicate is at positions 335–342 and 411–418 (**b**) (gray). Short motifs are possibly conserved/inserted/repeated/duplicated among CHIKV strains; the (V/A)S(M/T) and TSL are at positions 334–336 and 338–340 (**a**) and 410–412 and 414–417 (**b**) (orange boxes). Interestingly, these motifs are located in the *Ae. aegypti* inserted S(M/T)(T/I)TSLTH motif.

The CHIKV RNA genome is relatively conserved among the various lineages. However, its nsP3 has 6% differences even among closely related isolates [236]. It is well accepted that deletion events in nsP3 HVD are generally well-tolerated by alphaviruses [237]. For CHIKV nsP3 HVD, previous studies have found 4 and 7 aa deletions as well as 76 aa duplication events within its nsP3 HVD *N*-terminal [237,238,239,240,241]. CHIKV nsP3 mutation events are focused in the 326–524 aa position in the HVD region [79,185]. Interestingly, based on previous findings, all nsP3 HVD mutation events occurred in approximately the same region and started at position 376 for the 7 aa deletion and 76 aa duplication and at position 379 for the 4 aa deletion (Figure 2). Below is a list of CHIKV strains corresponding to the aforementioned mutation events (Table 1).

Interestingly, four human patient isolates (MY002IMR/06/BP (accession no: EU703759), MY003IMR/06/BP (accession no: EU703760), MY019IMR/06/BP (accession no: EU703761), and MY021IMR/06/BP (accession no: EU703762)) and four non-human primate isolate (M125 (accession no: KM923917), M127 (accession no: KM923918), M128 (accession no: KM923919), and M129 (accession no: KM923920)) which all were isolated from the outbreak in Bagan Panchor, Malaysia, in 2006 [241] were identified not to have any aa deletion in their nsP3 HVD [250].

To date, researchers are still unsure of the contributions provided by deletion events to any CHIKV fitness advantage which may have allowed the deletion to be maintained in the Asian lineage [245]. A previous study suggested that the nsP3 HVD 7 aa deletion is not an occasional event caused by viral culture in vitro, since it has been identified in many isolates from different regions [241]. Interestingly, CHIKV strains isolated from Malaysia have adapted more mutations than other strains from other locations [251]. This includes the nsP3 HVD 7 aa deletion event, which was found only in human isolates MY/06/37348 and MY/06/373750, and not monkey isolates M125, M127, M128, and M129. Remarkably, both the human and monkey isolates were from the same Bagan Panchor clade. A study suggested that minimal or no genetic changes at all were required for the virus spillover into a new host [252]. Thus, it has been suggested that a small genetic difference, such as an nsP3 HVD 7 aa deletion event, might help in the transmission and adaption of CHIKV from a human to a monkey host as its new reservoir [250,252].

Prior to 2006, no CHIKV (Asian) isolate, particularly in Malaysia, had been identified to have the 4 or 7 aa deletions at similar nsP3 HVD positions [237,239,241]. Thus, the deletions are thought to represent a recent evolutionary change in the Asian lineage [237,241]. It was proposed that the initial 4 aa deletion only involved (V/T)HTL residues; however, the CHIKV (Asian) dissemination from Indonesia to Malaysia resulted in a loss of three more codons [237], producing the 7 aa deletion (V/T)HTL(P/I)(S/H)T (Figure 2). It was suggested that the 4 aa deletion at position 379–382 and the 7 aa deletion at position 376–382 might have occurred independently in the Indonesian B3 clade and Malaysian B2 clade, respectively. The deletions arose as the result of CHIKV (Asian) evolutionary adaptation to the specific local setting; for example, the CHIKV (Asian) MY/06/37348 and MY/06/37350 were isolated during a local CHIKV outbreak in Bagan Panchor Perak, Malaysia, in 2006 [225,237,250]. In addition, based on the human eukaryotic linear motif (ELM) predictions, it has been suggested that the deletion motifs might play a role in phosphorylation and virus stability [242]. However, the biological consequence(s) of the nsP3 HVD deletion event still need to be identified [237], and wet lab experiments are needed to deepen our understanding of the role of these deletion motifs in both alphaviruses and their hosts [242].

Notably, the 4 or 7 aa deletion motifs and the whole 76 aa duplicate motif do not occur within or affect any CHIKV nsP3 motifs, which have previously been identified to be important in virus–host interactions. However, CHIKV is a re-emerging arbovirus and has a mix of virus populations with different genomic variants (quasi-species). These variants have plasticity characteristics and are able to adapt to new environments [242,253]. For example, one study showed that 239 aa pairs of CHIKV nsP3 have co-evolved and are proposed to share common functions [242], as described in Table 2. The aa co-evolution has allowed the virus to establish preferred characteristics, leading to its increased fitness in quasi-species environments. In that study, it was shown that the existence of HVD at its C-terminal domain allowed nsP3 to achieve the highest co-evolving residue number, with a total of 27, when compared to other non-structural proteins (nsP4 (18), nsP2 (16), and nsP1 (13)).

A recent study divided the CHIKV nsp3 HVD into A, B, C1, C2, C3, and D fragments (Figure 2). As demonstrated in earlier studies, all deletion and duplication events occur at fragment A, while fragment B consists of P-rich region (Figure 2). Fragment B is where interactions occur between the P-rich region and cellular host proteins consisting of the SH3-binding motif, such as the Bin1/amphyphisin2/SHSP9 and A0A182G3T6 (mosquito homolog of BIN1) (Figure 1c). Notably, fragments B and C1 consist of the PMASVR motif (Figure 2), which is assumed to interact with the CD2AP and SH3KBP1 proteins [12] (Figure 1c). In addition, fragments B, C1, and C2 contain the aa motifs for interacting with the FHL1 and FHL2 host proteins (Figure 1c). Likewise, fragment C3 was suggested to have aa motifs for interactions with members of the NAP1L family and has a low affinity toward G3BP family proteins. Finally, fragment D was demonstrated to interact with G3BP family proteins via FGDF-like motifs [2] (Figure 2). Table 2 lists the CHIKV nsP3 aa residues involved in co-evolution and related details.

### 6.2. Indel, Repetition and Duplication Events in AURAV nsP3

AURAV BR/P05 (accession no: MG761767), a new AURAV isolate, was discovered by Mosimann et al. 2018 [254], from the fifth passage of contaminated cell culture, which was intentionally used to propagate a confirmed human DENV-3 clinical sample. It was first discovered based on phenotype observation after an increased virus titer was detected from a C6/36 insect cell, yet no virus growth was detected in the Huh.7.5 human cell line when infected by a DENV-3 clinical sample. Interestingly, AURAV is not pathogenic to humans, and no vertebrate host for AURAV has yet been identified. This fact is in parallel with the observation that BR/P05 can only propagate in C6/36 and not in Huh.7.5 cell lines. The same condition has been identified for another insect-specific alphavirus, the Eilat virus (EILV). It was concluded that EILV could not propagate in mammalian cells due to the inability of its structural proteins to initiate efficient attachment and entry into the vertebrate host cell [255]. Mosimann et al. could not trace how BR/P05 came to exist in the DENV-3 clinical sample, and suggested that it might possibly have been due to contamination from the previous lab before they received the clinical sample.

**Figure 3:**
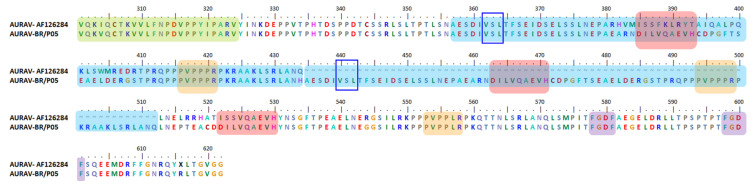
Important motifs and mutation events in AURAV nsP3 HVD. The HVD starts at position 325. It has two P-rich regions at positions 416–421 and 553–558 (peach) as well as two FGDF-like motifs at positions 578–581 and 598–601 (purple). There is a duplication motif for BR/P05 at position 435–512, with a motif sequence template from 357–434 (blue). There is also a repetitive motif, DILVQAEVH, at positions 385–393 and 522–530 as well as one duplication motif at position 463–471 (red). There is also a short motif, VSL, that is possibly conserved and duplicated among AURAV strains at positions 362–364 and 440–442 (blue boxes).

Isolated AURAV BR/P05 was found to have a high percentage of similarity in its genetic properties, at 92.9% for non-structural ORF and 96.6% for structural ORF compared to the previously reported AURAV isolate, AF126284. As shown in Figure 3, both AURAV strains have two P-rich regions at positions 416–421 and 553–558 (peach), as well as two FGDF-like motifs at positions 578–581 and 598–601 (purple). The only differences between both strains are (i) the 78 aa duplication motif and (ii) the substitution of the opal stop codon with arginine; both are unique characteristics present in the BR/P05 strain.

Based on the Weaver et al. study conducted on EEEV [256], Mosiman et al. hypothesized that the existence of the 78 aa duplication and the opal stop codon substitution with arginine were due to long exposure and adaptation to the insect cell line and, in this case, most probably the C6/36 cell line. nsP3 HVD has been determined to play roles in the assembly and formation of specific virus–host complexes [162]. Therefore, the 78 aa duplication motif in BR/P05 HVD is suggested to be involved in the adaptation of AURAV to different hosts. It was suggested that AURAV uses the 78 aa duplication for adaptation via a few different mechanisms, including by providing the addition of one P-rich region motif at position 494–499. The P-rich region has previously been shown to interact with host proteins consisting of the SH domain, such as amphiphysin 1 and 2 [79]. The 78 aa duplication region is also suggested to play a role in influencing nsP3 interaction with either cellular hydrophobic residues, host membranes, or in combination [37], based on differences in this region observed in a hydrophobicity plot. Interestingly, Mosimann et al. determined that there were triplicate DILVQAEVH motifs in BR/P05 HVD, including one in the duplication motif at position 463–471 (Figure 3), the function of which is yet to be discovered. These findings have strongly pointed to the roles of HVD in adapting alphavirus replication mechanisms based on the virus–host specific mode.

### 6.3. Indel, Repetition and Duplication Events in SINV nsP3

SINV is an alphavirus prototype [130], and many studies have been conducted in order to understand alphavirus biology based on SINV’s biological mechanisms. Several SINV strains such as S.A.AR86 (accession no: U38305), Girdwood S.A (accession no: U38304), and Ockelbo82 (accession no: M69205) have been shown to have indels in their nsP3, particularly in nsP3 HVD when compared to AR339 (accession no: J02363); the first SINV prototype was isolated from the *Culex* sp. mosquito pool in Egypt [206,257]. Both the S.A.AR86 and Girdwood S.A strains are South African isolates [258,259], and their genetic properties are more similar to those of strain Ockelbo82, which was isolated from Sweden, as compared to AR339. Only Girdwood S.A was isolated from a human patient, while AR339, S.A.AR86, and Ockelbo82 were isolated from mosquitoes from Culex sp. S.A.AR86, Girdwood S.A, and Ockelbo82 have all been associated with human disease [206].

**Figure 4:**
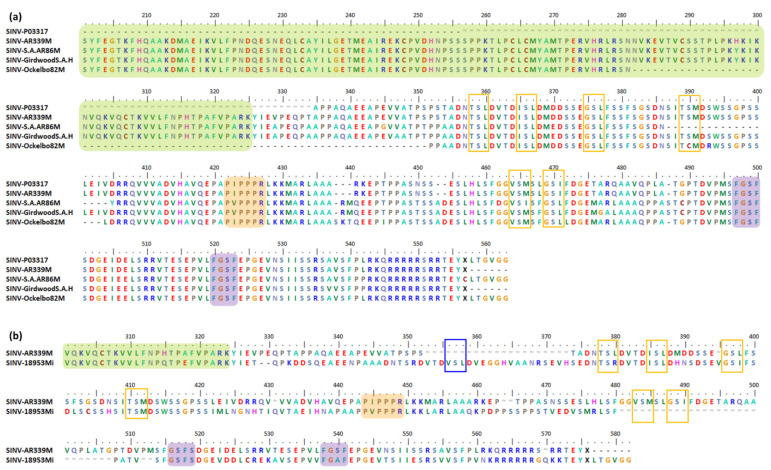
Important motifs and mutation events in SINV nsP3 HVD. The HVD starts at position 325. It has a P-rich region at position 422–427 (**a**) or 444–449 (**b**) (peach), and two FGDF-like motifs at positions 497–500 and 520–523 (**a**) or 516–519 and 538–541 (**b**) (purple). In (**a**), the Ockelbo82 has a 70 aa deletion at position 282–351, from AUD (green) to HVD (white). Meanwhile, S.A.AR86 and Ockelbo82 have 18 and 3 aa deletions at positions 387–404 and 401–403, respectively, which overlap at 401–403. There are 3 and 2 aa insertions in Ockelbo82 and both S.A.AR and Girdwood S.A at positions 438–440 and 439–440, respectively. At position 458–459, Ockelbo82, S.A.AR, and Girdwood S.A have a 2 aa insertion, while at position 487, S.A.AR86 and Girdwood S.A have a small 1 aa insertion. In (**b**), the SINV SINV_AUS_1975_18953 is shown to have a 21 aa insertion and a 27 aa deletion at positions 353–373 and 481–507, respectively. There is also a conserved short motif, the VSL, at position 356–358 (**b**) (blue box), which is possibly inserted in SINV_AUS_1975_18953. A few predicted VSL motifs such as TS(L/R), ISL, GS(L/I), and (T/V)(S/C)(M/I) have been conserved/inserted/deleted/repeated in SINV strains as well (**a** and **b**) (orange boxes).

As shown in Figure 4, several important motifs and indels have been determined. All five strains have P-rich regions (peach) and FGDF-like motifs (purple). Interestingly, only S.A.AR86 has a cysteine at position 557, while the others have the opal stop codon. Conversely to the Egyptian prototype AR339, the Girdwood S.A does not have any aa deletions. However, both S.A.AR86 and Ockelbo82 have 18 and 3 aa deletion events, overlapping each other for 3 aa residues. In addition, the Ockelbo82 has a very long 70 aa deletion from AUD to HVD. Regarding insertions, S.A.AR86, Girdwood S.A, and Ockelbo82 all have small insertions at nearly the same positions. For example, Ockelbo82 has a 3 aa insertion, while both S.A.AR and Girdwood S.A have a 2 aa insertion. All three strains have a 2 aa insertion at position 458–459, while only S.A.AR86 and Girdwood S.A have a small 1 aa insertion at position 487 [206,257].

Previous studies have demonstrated the association of the S.A.AR86 18 aa deletion and its cysteine substitution in place of the opal stop codon position, besides other factors in SINV E1 and E2 glycoprotein, with determining the neurovirulence of S.A.AR86 in adult mice [54,112].

Notably, a strain from Australia, SINV SINV_AUS_1975_18953 (accession no: MG182396), isolated from suckling mice from the *Culex annulirostris* pool in 1975, also showed indels in its nsP3 gene (Figure 4b). The indels include a 21 aa insertion and a 27 aa deletion [260]. This SINV isolate has a 95.1% similarity with a Malaysian isolate, MRE-16 (accession no: AF492770 and U90536). However, the significant impacts of those indels in SINV_AUS_1975_18953 and Ockelbo82 with a very long 70 aa deletion have yet to be determined.

### 6.4. Indel, Repetition and Duplication Events in SFV nsP3

Besides SINV, the SFV is also an alphavirus prototype. Since it was first isolated from a female mosquito pool in Bwanda, Uganda, in 1942 [261,262], it has been used as a model for arbovirus laboratory studies [262]. Its mammalian hosts are small rodents [116].

**Figure 5:**
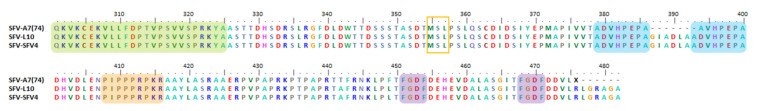
Important motifs and mutation events in SFV nsP3 HVD. The HVD starts at position 325. It has an extended P-rich region at position 408–416 (peach), and two FGDF-like motifs at positions 451–454 and 468–471 (purple). A7(74) has a 7 aa deletion at position 387–393. The duplicate motif ADVHPEPA flanks the GIADLAA deletion motif at positions 379–386 and 393–400 (blue). A predicted conserved motif, MSL, can be observed at position 355–356 (orange box).

As shown in Figure 5, the SFV has an extended P-rich version (peach) [3] and a duplicate FGDF-like motif (purple). It has been demonstrated to tolerate 43 to 119 aa deletions in its nsP3 HVD, with a slight decrease in its in vitro replication and virulence in mice [124]. For example, SFV A7(74) (accession no: Y12518) has a 7 aa deletion of GIADLAA motif in its nsP3 HVD as compared to the SFV L10 (accession no: AY112987) and SFV4 (accession no: AJ251359) strains. The A7(74) is an avirulent and asymptomatic strain isolated in 1959 in Mozambique [263], and its severity strongly depends on the host age. It is only lethal toward two-week-old neonatal mice [264,265]. This characteristic is most probably due to the ability of A7(74) to form a virion in propagating neurons and not in mature neurons [265]. However, both L10 and SFV4 that retain the GIADLAA motif are virulent strains and could cause lethal encephalitis and death in mice of all ages within a few days [266,267].

The deleted GIADLAA motif, which consists of five hydrophobic residues [116], has been observed in various host proteins. Thus, it has been suggested to be part of the host RNA and inserted into the SFV nsP3 HVD genome [37]. In addition, the deletion motif in SFV A7(74) is flanked on both sides by ADVHPEPA motifs (blue) (Figure 5). Interestingly, the ADVHPEPA motif was observed to exist at the same position in virulent strains L10 and SFV4 (Figure 5) [116].

Initially, SFV nsP3 was demonstrated to assemble on the surfaces of host cellular vacuoles. Thus, it was suggested that nsP3 is a docking protein which stabilizes the active viral replication complex on host cellular vacuole surfaces [268,269]. Subsequently, Tuittila et al. (2000) attempted to demonstrate that the deleted motif in SFV nsP3 HVD may be involved in this mechanism. It was suggested that a reduction in the nsP3 HVD hydrophobicity might occur via a HVD deletion event, causing reduced interaction between nsP3 HVD and cellular vacuole surfaces and lower efficiency of SFV RNA synthesis. However, the study found that the reconstruction of the GIADLAA motif at the rA774 deletion position did not increase the clone’s virulence in causing clinical symptoms, nor differentiate it from other avirulent clones or even its rA774 parent. The study confirmed that changes in its nsP3 HVD length and sequences do not affect the SFV phenotype [116].

### 6.5. Indel, Repetition and Duplication Events in RRV nsP3 HVD

RRV has caused the highest rate of mosquito-borne human infection in Australia. Until 2020, approximately 5000 clinical cases of RRV infections have been recorded annually [136]. The RRV virgin soil epidemic in 1979–80 infected approximately 500,000 people in Pacific Island Countries and Territories (PICTs) [270]. It was suggested to be caused by a viremic traveler in Fiji and expanded to surrounding areas via a human–mosquito–human transmission cycle, since their macropod hosts did not exist in PICTs [271,272]. Generally, RRV has a sylvatic cycle among various mosquito vector species and its ideal vertebrate host—namely, kangaroos and wallabies [273,274]. Humans are a dead-end host as RRV infection usually leads to unsuccessful transmission due to its low titer and short-lasting viremia [275]. By infecting the human population, it causes pain and suffering to patients and causes a burden on the Australian economy [136]. This virus is highly dependent on external factors in order for it to successfully infect and transmit. An ideal environment and climate will influence reservoir populations and mosquito vectors and provide warmer months [276].

Based on a thorough genome-scale phylogenetic and evolutionary analysis from 2020, it was suggested that four RRV lineages exist in Western Australia—namely, North-Eastern (G1), Western (G2), Eastern (G3), and Contemporary Western (G4). In the last 50 years, RRV has emerged in new lineages every decade. Based on this study, it was found that the G2 and G4 lineages are most related to the G3 lineage. Interestingly, the G3 lineage subsequently replaced the G1 and G2 lineages after the PICT epidemic. However, the G4 lineage has become the dominant lineage in Australia since its discovery in 1994. Nowadays, both G1 and G2 lineages are under detection due to their low circulation or having gone extinct [136].

**Figure 6:**
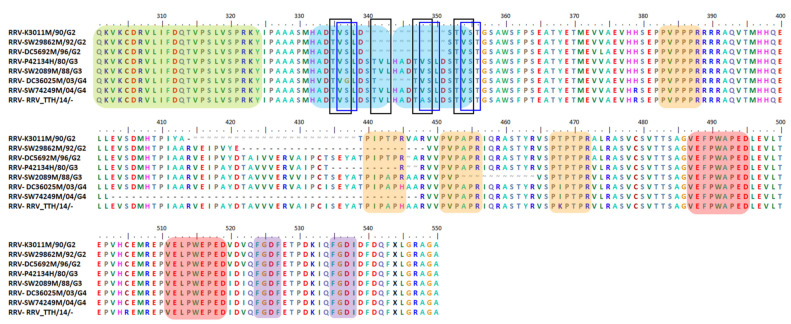
Important motifs and mutation events in RRV nsP3 HVD. The HVD starts at position 325. It has four P-rich regions at positions 383–388, 440–445, 451–456, and 467–472 (peach) as well as two FGDF-like motifs at positions 524–527 and 535–538 (purple). There are 1–45 aa deletions, starting at position 403–464, which mostly abolished half of or the entire second or third P-rich regions of the selected strains, such as SW29862, P42134, SW2089, and SW74249. It also has duplication motifs of VE(F/L)PW(A/E)PED at positions 487–495 and 511–519 (red), and the HADT(V/A)(S/G)LDSTV(L/S) duplication motif in blue boxes at positions 332–343 and 344–355 (blue). Meanwhile, another study suggested that despite having duplication events at positions 332–343 and 344–355, there was a 12 aa insertion in the STVLHADT(V/A)SLD at position 340–351. Notably, there are four duplicates of T(V/A)(S/G/L) at positions 335–337, 341–343, 347–349, and 353–355 (black boxes). There is also a short motif that is possibly conserved/inserted/repeated among RRV strains, (V/A)(S/G)(L/T), at positions 336–338, 348–350, and 354–356 (blue boxes).

As shown in Figure 6, a repetition of four P-rich regions (peach) and two FGDF-like motifs (purple) has been observed to exist in RRV nsP3 HVD [37,136]. In addition, a recent study detected multiple interesting deletion and duplication events in their nsP3 HVDs. For example, 24 isolates from the G2, G3, and G4 lineages were found to consist of 1–45 aa deletions of their nsP3 HVD [136]. Notably, 22 of the deletions have demolished half or all of the second or third P-rich regions, without interfering with the existing FGDF-like motifs, as may be seen for strains SW29862 (accession no: MN038271), P42134 (accession no: MN038252), SW2089 (accession no: MN038260), and SW74249 (accession no: MN038282). Interestingly, out of 24 isolates, only one strain was isolated from humans—the P42134 (accession no: MN038252–1980)—while other strains were isolated from various mosquito species. Two isolates from the G2 lineage, RRV K3011 (accession no: MN038221–1990) and DC5692 (accession no: HM235643–1996), have deletions, but none of their four P-rich regions are disturbed [136].

Interestingly, Aaskov et al. (2011) determined the duplication motifs of VE(F/L)PW(A/E)PED in RRV nsP3 HVD (Figure 6) [37]. In addition, the same study and a more recent study suggested that a duplication or insertion event involving 12 aa residues occurred in RRV nsP3 HVD, approximately at the same positions toward the *N*-terminal of HVD [37,136]. The duplication or insertion motifs were only observed in RRV isolates from 1979, concurrent with the PICT virgin soil epidemic until now, which means that only G3 and G4 lineages have them, while the G1 and G2 isolates do not. The motifs were the insertion of STVLHADTVSLD [136] or the duplication motifs of HADTVSLDSTVS/L [37,135] (Figure 6). Notably, Aaskov et al. (2011) also suggested that TVS motifs within HADTVSLDSTVS/L duplicate regions are repeated four times in a less conserved form (black boxes) (Figure 6), and they might be marks from previous duplication events in RRV nsP3 HVD. In addition, from our observations, these suggested insertion or duplications motifs from Michie et al. (2020) and Aaskov et al. (2011) overlap with each other at the HADTVSLD residues at position 344–351. Interestingly, they also observed 5 aa deletions within the 12 aa insertion/duplication at position 342–346, which occurred in DC36025 (accession no: MN038209), a mosquito isolate. The 5 aa deletion caused the insertion motif to change from STVLHADTVSLD to STTVSLD (Figure 6). Notably, the sequence for RRV_TT (accession no: KY302801) was updated on 23rd July 2020. Due to this, no more 12 aa deletion, as mentioned by Michie et al. (2010), was observed within its nsP3 HVD.

The previous study demonstrated that the duplicate region HADTVSLDSTVS/L stabilized the mildly disordered nsP3 RNA structure into a more stable stem-loop [37]. The STVLHADTVSLD or HADTVSLDSTVS/L insertion/duplication event was initially suggested to contribute to RRV fitness advantage, causing the average number of cases of RRV per year in Australia to increase from 500 in 1980 to 5000 in 2018. Interestingly, a steady replacement of G1 and G2 lineages by G3 also happened concurrently with the case number increment. In relation to that, the duplication event was suggested to be one of the reasons why G3 was able to replace G1 and G2, possibly alongside other nucleotide polymorphism(s) [37]. Despite this, the current study takes a different perspective of the insertion/duplication’s impact proposal, as RRV disease has become noticeable after the PICT epidemic. Thus, the acknowledgement by the authorities has led to an increasing number of RRV cases being reported. Moreover, the 12 aa insertion/duplication in G3 and G4 lineages was observed in 1968, which was approximately 11 years before the PICT epidemic, and the G2 lineage was circulating for 15 years before the G3 lineage was first detected. Hence, Michie et al. (2020) proposed that it is unlikely that the insertion event played a crucial role in the fitness of the PITC epidemic [136].

Hence, we still remain unsure of the role(s) and function(s) of i) the deletion event, which mainly interfered with one of the RRV P-rich regions, and ii) the insertion/duplication and deletion within insertion/duplication events, which likely conserve the TVSLD motif. Previous studies have suggested that investigations need to be performed in order to determine the significance of these events.

### 6.6. Indel, Repetition and Duplication Events in BFV nsP3 HVD

BFV is an endemic arbovirus in Australia. Around 1000 cases are reported per year, and the virus causes a disease with symptoms very similar to those of RRV. BFV was first isolated from the *Culex annulirostris* pool in 1974 and was associated with human disease in 1988 [277,278,279]. Since then, it has caused a few outbreaks in Australia [186,280,281,282] and was detected in Papua New Guinea [283]. However, BFV is considered more stable and has emerged with new lineages less frequently than RRV has [186]. A previous study on BFV genome-scale phylogenetic analysis using 34 mosquito isolates in a 44-year period from Australia and Papua New Guinea classified BFV into three lineages: G1, G2, and G3 [186]. Based on these analyses, the BFV nsP3 was found to be more conserved than RRV, with 99% average pairwise nucleotides and an aa length 1.2 times shorter than that of RRV and CHIKV.

**Figure 7:**
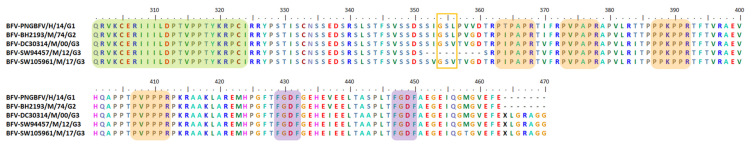
Important motifs and mutation events in BFV nsP3 HVD. The HVD starts at position 325. It consists of four P-rich regions at positions 363–368, 373–378, 387–392, and 407–412 (peach) as well as two FGDF-like motifs at positions 429–432 and 447–450 (purple). The SW94457 has a 9 aa deletion motif at position 352–350. A predicted conserved motif for GS(L/V) has been observed at position 355–356 (orange box).

As shown in Figure 7, BFV has four P-rich regions (peach) and two FGDF-like motifs (purple). In addition, a deletion consisting of 9 aa was identified in one of the isolates, BFV SW94457 (accession no: MN689044). This corresponds to a hydrophobic region consisting of (I/V)GS(V/L)(T/P)VGDT residues [186]. The study also identified that BFV has fewer indel events in nsP3 but many large indels in its 3′ UTR, while RRV was identified to have many large indel events in its nsP3, though with fewer and smaller indels in its 3′ UTR [186]. It has been suggested that the unique characteristics of BFV and RRV nsP3, alongside their 3′ UTR, could influence their interactions with a range of hosts [94,186,284]. Even though it is assumed that BFV and RRV use the same mosquito vector and vertebrate host [285,286], a preliminary study has suggested that RRV-infected mosquitoes are more infectious than BFV-infected mosquitoes, due to RRV’s significantly faster and higher rate of replication and more persistent titer compared to BFV. Hence, it was suggested that unique characteristics of the nsP3 and 3′ UTR might be associated with the different transmission dynamics of both BFV and RRV [186].

As discussed above, several important motifs which are involved in alphavirus fundamental mechanisms have been identified in alphavirus nsP3 HVD, such as the FGDF-like motif. The motif is conserved in duplicate form for the most alphaviruses, except for MAYV, EEEV, VEEV, and WEEV. MAYV and EEEV were identified to have only one FGDF-like motif in their nsP3 HVD, while none were observed for VEEV and WEEV. Another motif, the P-rich region, is also conserved among alphavirus. It even exists in repetitive forms in several species, such as being replicated four times in RRV and in duplicate form for VEEV, EEEV, and WEEV. In general, mutation events such as indel, repetition, and duplication might be caused by an antigenic drift due to a lack of RdRp proofreading activities, and an antigenic shift due to the recombination and reassortment of viral genomes [97]. The insufficient RdRp activities were suggested to occur during negative-strand RNA synthesis, as the nsP4 RdRp might tend to switch the RNA template when associated with uncleaved nsP123 polyprotein. The nsP4 RdRp was proposed to have better associations with nsP1, nsP2, and nsP3 proteins during ss(+)RNA synthesis [37]. An example of this phenomenon may be observed in AURAV BR/P05. It has been speculated that its 78 aa duplication motif resulted from an replication error or homologous recombinant [287], with some contributions from additional events [254].

The aa insertion, repetition, or duplicate events in alphavirus nsP3 HVD have been suggested to be formed by copying from other regions of nsP3, as we could see for P-rich region, the FGDF-like motif, and other repetitive or duplicate motifs in CHIKV MUM09-Selangor-2009 (Figure 2) [238], AURAV BR/P05 (Figure 3) [254], and RRV duplicated motifs (Figure 6) [37]. The mutation events could also be caused by the copying of random foreign genetic material, particularly host cellular proteins, as seen in CHIKV 06-021 (accession no: AM258992), where its STITSLTH motif within the STITSLTHSQFDLSVDGE insertion is identical to part of the sequence for a putative zinc finger protein from *Ae. aegypti* (Figure 2). The same observation was also found in SFV when its GIADLAA motif was found in various host proteins [37]. The insertion of foreign RNA into nsP3 has also occurred in VEEV [288,289,290], EEEV, and SINV, as previously discussed [37]. We propose that these various mutations are part of a strategy to allow alphavirus to replicate sufficiently and survive through existing selective pressures, especially throughout the repetitive bottleneck events during transmission between mosquitoes and vertebrate hosts.

There are several putative purposes and functions of nsP3 HVD indel, repetition, and duplication events that might contribute to the alphavirus host-specific mode—for example, by providing more/less/none nsP3 HVD, important interacting motifs with redundant functions, e.g., different numbers of P-rich regions among alphavirus species [3]. The mutation events also may provide more/less nsP3 HVD putative phosphorylation sites via duplication or deletion events, such as in the nsP3 HVD *N*-terminal of CHIKV MUM001–2009-Selangor (accession no: KX168429) [238] and SINV S.A.AR86 [206], respectively. In addition, they have also been suggested to provide stability for the nsP3 RNA structure. For example, the RRV F9073 duplicate region HADTVSLDSTVS/L stabilizes a mildly disordered nsP3 RNA structure into a more stable stem-loop [37], while a 34 aa duplicate region has been demonstrated to strengthen the predicted large stem-loop structure for VEEV [288]. In addition, ability of nsP3 HVD to tolerate indel, repetition, and duplication events has allowed it to have a certain characteristic plasticity while interacting with various distinct host cellular proteins. For example, a duplication consisting of 33 aa in VEEV HVD was suggested to lead to an efficient VEEV replication in BHK-21 cells and was correlated with an increasing level of pathogenicity in humans [171]. Moreover, the viral RNA template length is closely related to viral RC spherule size, as shown for SFV [291]. RC spherules gather necessary components, including nsP4 RdRp, other alphavirus nsPs, and possibly host cellular proteins, to be structurally arranged within. This mechanism is essential, as it has been suggested to produce different alphavirus RNA species and allows us to understand how alphavirus RCs switch the RNA strand specificity [179,292]. Therefore, we believe that the nsP3 HVD plasticity characteristic involving indel, repetition, and duplication events has an important role in determining the size of RCs spherules and is also involved in gathering specific host cellular proteins to the RCs spherules according to its specific nsP3 HVD aa sequence interactions with distinct host cellular proteins. Interestingly, the plasticity characteristic is not just restricted to alphavirus nsP3 HVD; several motifs in SINV mRNA have also demonstrated functional plasticity via evolution in the adaption to different hosts and environments. It was suggested that alphavirus was firstly transmitted from marine vertebrates to insects, which later became an effective alphavirus transmission vector for infection among land vertebrates. Alphaviruses were proposed to recruit a motif at their 3′ UTR for efficient translation during their adaptation to the insect host [32]. The duplication of 78 aa in AURAV BR/P05 nsP3 HVD has been found to make a difference in its hydrophobicity plot [254], while RRV repetitive P-rich and VEFPWAPEDL motifs, together with their occurred variation motifs, are usually hydrophobic [37,254]. Based on their locations near to the HVD C-terminal and hydrophobic-related characteristics, these duplicate motifs were suggested to either influence the interactions between nsP3 and host membranes, membrane-like structures, other molecules consisting of hydrophobic residues, or in combination [37,254], as was previously suggested for SINV nsP3-containing complexes [86]. In addition, the SFV A7(74) strain’s GIADLAA deleted motif was observed to consist of five hydrophobic residues [116]. The deletion has been suggested to cause fewer hydrophobicity characteristics in nsP3, thus reducing the nsP3-host membrane vacuole-binding properties, such as during the stabilization of viral RC cytopathic vacuoles and also during nsP3 accumulation on the surfaces of host vacuoles [268,269]. This phenomenon has also been suggested to affect viral RNA transcription efficiency [116]. Interestingly, the nsP3 HVD *N*-terminal of BFV SW94457 (accession no: MN689044) also has a deletion event in the hydrophobic region consisting 9 aa residues, which occurs at position 352–350 (Figure 7) [186]. Whether this region is associated with BFV nsp3 interactions with host membranes or other hydrophobic molecules was not discussed.

Currently, the accumulating data point to the existence of a putative relation between various alphavirus nsP3 HVD indel, repetitive, and duplication events and virus–host specificity and phosphorylation activity. For example, the deletion of 18 aa, which consists of seven serine residues, has been suggested to affect the overall phosphorylation of SINV S.A.AR86 nsP3 [112], while the 78 aa duplication motif in AURAV BR/P05 has been suggested to be involved in the HVD phosphorylation process [254].

Phosphorylation is a protein post-translational modification mechanism that is important in essential functions, such as protein–protein interactions, protein folding, intracellular localization, signal transductions, and transcriptional regulation as well as in processes involved in cell cycle development, viability, and apoptosis. It is a piece of host machinery which is partly or fully hijacked by intracellular pathogens, allowing them to establish an efficient infection cycle. Protein phosphorylation is achieved by adding a phosphate group to serine (S), threonine (T), and tyrosine (Y) using adenosine triphosphate (ATP) [293].

Among the four alphavirus nsPs, only nsP3 is phosphorylated [294,295]. It was previously shown that nsP3 HVD consists of numerous phosphorylation sites as it is a domain that is rich in serine and threonine residues [121,162,294,295]. Interestingly, the tyrosine residue was found not to be phosphorylated, while serine was found to be more phosphorylated than threonine [269,295,296]. The highly variable or less conserved characteristic of nsP3 HVD greatly affects the number of phosphorylation sites in different alphavirus species, and various nsP3 HVD phosphorylation conditions have been observed during alphavirus infection [89,294,295]. The phosphorylation of serine and threonine was suggested to be conducted by multiple cellular host kinases [7,161,269]. For example, casein kinase II (CK2) along with other kinases such as protein kinase C (PKC) have been suggested to contribute to SINV and SFV nsP3 phosphorylation [295,296]. In addition, the VEEV and WEEV nsP3s have demonstrated interaction with host inhibitor of nuclear factor kappa-B kinase subunit beta (IKKβ) [297]. Interestingly, the alphavirus nsP3 macrodomain has been an important site for targeting kinase recruitment [122]. The nsP3 HVD was suggested to be phosphorylated during the early replication stage via post-translational modification [118]. In addition, the nsP3 in the P15 membrane fraction is more heavily phosphorylated than nsP3 in the S15 cytosolic fraction [294].

The role(s) of phosphorylated nsP3 HVD is still unclear, but it was required for optimal SINV and SFV RNA synthesis depending on the host type [89,122,295,298]. It was demonstrated that up to 16 serine and threonine positions of SFV and SINV could be phosphorylated [295,296,298], and that SINV is heavily phosphorylated when compared to SFV [294,295]. For SINV nsp3, it has been demonstrated that phosphorylation is required for synthesizing the negative-strand RNA and SG RNA due to its existence at the early stages of alphavirus infection [295,298,299]. Phosphorylated nsP3 HVDs, particularly the serine and threonine residues, were suggested to be involved in the attachment of alphavirus SINV RCs to the host cell’s cytoskeleton [85,86]. Meanwhile, previous studies have shown that SFV has phosphorylation activity within the 50 aa region of its *N*-terminal in nsP3 HVD [89,296]. It has been suggested that SFV nsP3 HVD phosphorylation contributes to its virulence, while mutated HVD has been demonstrated to interfere with nsP3 phosphorylation activity, therefore lowering the RNA synthesis level and greatly reducing the SFV virulence in mice [89]. In addition, VEEV was shown to have 53 potential phosphorylation sites [171]. Interestingly, the 34 aa deletion in its nsP3 HVD had an insignificant impact on viral infection in mammalian CEF, BHK-21, and Vero cells [288]. Furthermore, the nsP3 HVD phosphorylation activity was not crucial for VEEV propagation in the BHK-21 and NIH 3T3 vertebrate cell lines. However, the activity was suggested to be crucial for virus propagation in C710 insect cells [171]. A study by Teppor et al. in 2021 [177] demonstrated that, unlike SINV, SFV, and VEEV, the potential phosphorylation sites for CHIKV are not clustered at the nsP3 HVD *N*-terminal only but, rather, scattered throughout its nsP3 HVD. The substitutions of serine/threonine with alanine (A) at the *N*-terminal and C-terminal of nsP3 HVD have resulted in a reduction in CHIKV RNA synthesis and infectivity in mammalian cells, respectively. In addition, the same substitution throughout the whole nsP3 HVD has resulted in the full abolishment of CHIKV RNA synthesis and infectivity. However, the substitutions only significantly reduced the nsP3 HVD interactions with CD2AP, BIN1, and FHL1 proteins, while the interaction with G3BP1 protein was not affected. These findings on VEEV, SINV, and SFV are a strong indicator that nsP3 phosphorylation sites’ roles in viral replication mechanism regulation are dependent on the interactions between ranges of alphavirus species and host cell types [112,288].

Interestingly, the hyperphosphorylated nsP3 HVD of SFV and CHIKV has been demonstrated to induce the activation of the host’s phosphatidylinositol-3-kinase (PI3K)-protein kinase B (PKB or Akt) mammalian target of Rapamycin (mTOR), also known as the PI3K-Akt-mTOR pathway, which is a pro-survival signaling cascade [177]. This pathway activation was associated with the efficient internalization of SFV RCs from the cell periphery upon virus infection. The localization of RCs may affect pathology. However, the pathway activation was only moderately boosted by CHIKV, while during SFV infection the pathway boost was more potent and persistent [175,293]. It was proposed that alphavirus HVD phosphorylation sites have experienced rapid changes throughout alphavirus evolution [7], and these sites are suitable targets for inhibiting alphavirus replication [293].

Based on our observations, we notice that the indel and duplication events, involving a small or large number of residues, usually occur at the same aa positions, particularly among different strains from the same alphavirus species, as we could see at the nsP3 HVD *N*-terminal of CHIKV (Figure 2), SINV (Figure 4), RRV (Figure 6), and BFV (Figure 7). This finding agrees with the previous discussion on EEEV and SINV, where larger insertion motifs for different alphavirus lineages were always at the same sites [37]. Secondly, we noticed a few indel and duplication events involving long aa sequences from AUD to HVD regions for different alphavirus species. Interestingly, all of them consisted of approximately 70 aa residues, as we could see in the duplication of 76 aa in CHIKV MUM01–2009-Selangor (Figure 2), the duplication of 78 aa in AURAV BR/P05 (Figure 3), and the deletion of 70 aa in SINV SINV_AUS_1975_18953 (Figure 4).

**Figure 8:**
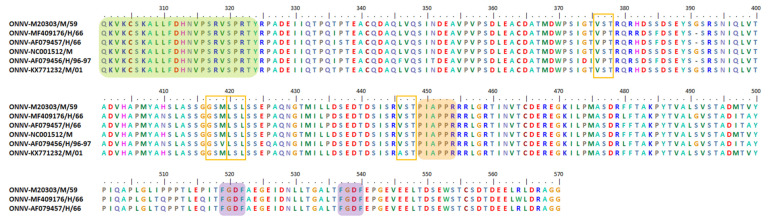
MSA and important motif locations in the nsP3 of selected ONNV strains. The HVD starts at position 325. It has one P-rich region at position 449–454 (peach) and two FGDF-like motifs at positions 519–522 and 538–540 (purple). The MF409176, AF079457, and AF079456 have 1 aa deletion at position 391. A predicted conserved motif, (V/A)(S/P)T, at positions 376–378 and 446–448; GS(M/V) at position 417–419; and LSL at 420–422 have been observed (orange box).

Thirdly, based on our observations in previous indel, repetitive, and duplication studies, we found out the VSL motif is potentially conserved, emerged by insertion, duplicated, or even deleted in various OW and NW alphaviruses. For example, the VSL motif, or its variations VGL, ASL, and VST, are observed in the previously discussed RRV-TVSLD motif at positions 336–338, 348–350, and 354–356 (Figure 6—blue boxes). The motif is repeated three times in RRV nsP3 HVD, conserved throughout the insertion/duplication, and deleted within the insertion of STVLHADTVSLD of RRV, as discussed previously. The VSL motif is also observed in AURAV BR/P05 [254] at position 362–364, duplicated at position 440–442 (Figure 3), or inserted in SINV SINV_AUS_1975_18953 [260] at position 356–358 (Figure 4b) and in EEEV NJ-60 (accession no: EF568607) at position 397–399 [37]. Based on MSA carried out between RRV and CHIKV strains, we managed to predict the VSL motifs in CHIKV, which are the (V/A)S(M/T) and TSL at positions 334–336 and 338–340, respectively (Figure 2—orange boxes). Interestingly, both motifs are part of the CHIKV STITSLTH insertion motif from the *Ae. aegypti* putative zinc finger protein [37]. Both motifs are also duplicated in CHIKV MUM001–2009-Selangor at positions 410–412 and 414–417 (Figure 2b—orange boxes). Based on the CHIKV potential VSL motifs, (V/A)S(M/T) and TSL, we were then able to find other similar motifs in SINV, such as TS(L/R), ISL, GS(L/I), (T/V)(S/C)(M/I) (Figure 4—orange boxes), and MSL at position 355–357 for SFV (Figure 5—orange box); (V/A)(S/P)T at positions 376–378 and 446–448, GS(M/V) at position 417–419, and LSL at 420–422 for ONNV (Figure 8—orange boxes); and GS(L/V) in BFV at position 354–356 (Figure 7—orange box). Interestingly, the GS(L/V) motif was deleted from BFV SW94457 [186]. As mentioned before for RRV, the repetitive TVS motif was proposed to be a trace of prior duplication events [260]. In addition, from the MSA that was conducted, the VSL motif and its variants were identified to be located at the *N*-terminal of alphavirus nsP3 HVD, which is known to be heavily phosphorylated [89,293]. Thus, we hypothesize that the S residue is conserved among alphaviruses and has the potential to be involved in phosphorylation activity for various alphavirus, as suggested for the 18 aa deletion event role in SINV HVD [112]. Interestingly, the VSL motif consists of only neutral and hydrophobic aa residues, thus potentially interacting with either the host membranes, other molecules of other cellular hydrophobic residues, or in combination [37].

Finally, we suggest that the existence of long duplication events of 78 aa in AURAV BR/P05 [254] and 76 aa duplication in CHIKV MUM001–2009-Selangor [238] might be associated with their co-existence with DENV-3 and DENV-2, respectively, in the same host cells. Previous studies have demonstrated that there are two types of virus mixed-infection interactions. The first type is due to enhanced replication and transmission of at least one of the viruses via the production of facilitative effects [300]. This type of interaction was demonstrated by the fact that *Culex* flavivirus (CxFV) was likely to infect the West Nile virus (WNV)-positive mosquito pools rather than the WNV-negative mosquito pools [301]. Furthermore, under laboratory co-infection conditions, WNV transmission was enhanced by CxFV [302]. It was suggested that CxFV might regulate or suppress mosquito immune recognition, thus allowing the mosquito to be more susceptible to infection by secondary pathogens [301]. Interestingly, the second type of mixed-infection interaction implicates a negative impact on the replication mechanism of the viruses involved, as was previously documented to occur among DENVs and also for DENV and CHIKV mixed infections. For example, the replication of all involved viruses was suppressed, or the replication of only one virus was enhanced, while others were suppressed [300]. The outcome of this type of interaction depends on the multiplicity of infection (MOI) of the infecting viruses and whether co-infection or super-infection occurred [303,304,305]. However, we cannot determine the virus MOIs from the reported studies, or whether the co-existences of AURA BR/P05 with DENV-3 and CHIKV MUM001–2009-Selangor with DENV-2 were a simultaneous co-infection or whether the host cells were infected at different times, thus allowing super-infection. A recent study hypothesized that virus replication mechanisms during mixed infection are characterized by the type of infected cells. The study also highlighted the potential intracellular resource competition between viruses during mixed infection, which could influence virus characteristics such as virulence, transmissibility, and resource division [300,306]. Thus, based on the aforementioned findings and the ability of CHIKVs and other alphaviruses to use a few different strategies for survival in host cells, such as having an opal stop codon in nsP3 to create a more persistent CHIKV infection in the host [125], we suggest that the existence of duplication events in both AURAV and CHIKV nsP3 HVD are an indication of the virus adaptation to their interactions with host cellular proteins to give them a suitable replication rate for survival and transmission during their co-existence with DENVs.

Besides highlighting the potential roles and functions of alphavirus nsP3 HVD indel, repetitive, and duplication events, our review has suggested several important issues to be considered when analyzing HVD mutation(s), such as whether the mutation is an isolated event or not; where and when it happens; whether it can be either fixed, removed, or both; and also the consequences of mutations for both the virus itself and its host—e.g., it could provide a fitness advantage or enable a change in virus epidemiology. We also have to consider the ability to pass the mutation on to the next generation [37].

## 7. Conclusions

To date, the nsP3 HVD has been recorded to bind to several distinctive individual host cellular proteins and has also been demonstrated to be involved in alphavirus replication, vector specificity, and virulence determination. It was also suggested to be a candidate regulator of host stress response and to act as an agent for alphavirus transmission between and among hosts.

However, we still have a poor understanding of these aforementioned mechanisms, both in relation to individual proteins or their involvement in co-factor protein networks or molecular-level pathways. This review highlights the putative involvements of alphavirus nsP3 HVD indel, repetitive, and duplication events in those fundamental viral mechanisms via virus–host specific mode characterization subsequently facilitating alphavirus evolution, viability, and emergence.

We believe that various selective pressures and repeating bottleneck events have resulted in changes to alphavirus nsP3 HVD—for example, (i) positively selected lineages, as we can see happening for RRV, where new lineages have periodically emerged approximately every decade, and (ii) positively selected codons (as we could observe in the events of the (a) conservation of important motifs, such as P-rich regions, FGDF-like motifs, and FXR-binding motifs; (b) the opal substitution sense codon; and (c) aa mutations, such as previously mentioned indel, duplicate, and repetitive events). The selective pressures from inside specific host types and environments will influence alphavirus species to be in a quasi-species condition, creating high variability in viral genomes among virus populations, particularly in the nsP3 HVD region, allowing adaptation. Thus, virus populations with selected mutations that facilitate virus survival in a specific host and environment will be further established and disseminated. Moreover, transmission into a new host—for example, from a mosquito to a vertebrate host—is a repetitive bottleneck selection event for alphavirus and could lead those viruses to evolve, survive, and emerge through the positive selection of codons.

A better understanding of nsP3 HVD roles in alphavirus fundamental mechanisms as well as their relation to the current information on the involvement of nsP3 HVD mutations, such as indel, repetitive, and duplication, in alphavirus evolution, viability, and emergence could enhance our understanding of alphavirus elemental characteristics. We believe that the nsP3 HVD mutation events deserve more of our attention in order to develop strategies to impede the alphavirus infection and transmission cycle. They are also suitable candidates to be considered in epidemic control measurements at the molecular level as a preparation for the inevitable future evolution and potential emergence of alphaviruses.

## Figures and Tables

**Table 1:** List of CHIKV strains with deletion or duplication events.

4 aa Deletion at Position 379–382			
Strain	GenBank Accession Number	Lineage	Details
Indonesia/0712aTW	FJ807886	Asian	All samples were taken from febrile patients who arrived at two Taiwanese airports from January 2006 to February 2009. Seven strains imported from Indonesia had 4 aa deletions in their nsP3 HVD. The Indonesia/0706aTW isolate shares a 99.42% genetic identity with MY0031MR, a CHIKV human isolate from Malaysia. MY0031MR was isolated in Bagan Panchor in 2006 [239,240].
Indonesia/0712bTW	FJ807887		
Indonesia/0802aTW	FJ807888		
Indonesia/0804aTW	FJ807889		
Indonesia/0806aTW	FJ807890		
Indonesia/0811aTW	FJ807891		
Indonesia/0706aTW	FJ807897		
14.02217	KY435477	Asian	The 14.02217 was isolated in 2014 from Guyana during an American CHIKV outbreak. It belongs to the Asian/American lineage [235].
PR-S6	KR264951	Asian	These four strains were isolated from South America in 2014: PR-S6 (Puerto Rico), 99,659 (British Virgin Islands), InDRE 51CHIK (Mexico), and InDRE 4CHIK (Mexico) [229,242,243].
99659	KJ451624		
InDRE 51CHIK	KP851709		
InDRE 4CHIK	KP851710		
Yap 13–2039	KJ689452	Asian	These four strains were isolated from Micronesia in 2013 from different hosts. The Yap 13–2039 was isolated from the *Ae. aegypti* mosquito pool, the Yap 13–2148 from the *Aedes hensilli* mosquito pool, and both 3807 and 3462 from human samples [229,242,244].
Yap 13–2148	KJ689453		
3807	KJ451622		
3462	KJ451623		
**7 aa Deletion Events at Position 376–382**			
MY/06/37348	FN295483	Asian	These strains are the earliest CHIKV strains isolated from Malaysia. They were isolated from a CHIKV outbreak in Bagan Panchor Perak in March 2006, which took place concurrently with the global ECSA strain outbreaks. The amplification and sequencing were conducted using original patient serum, confirming that the deletion event occurred during the outbreak of this virus strain and did not arise from laboratory passaging [216,217,237].
MY/06/37350	FN295484		
JMB-154	KX097982	Asian	JMB-154 was isolated during a DENV outbreak in Jambi, Indonesia, in 2015 from a patient with DENV-like illness symptoms. However, the patient was confirmed to be negative for DENV. The DH130003 was isolated from a traveler who returned to Germany after visiting Bali, Indonesia, in 2013 [241,245,246].
DH130003	KM673291		
SZ1239	MG664851	Asian	SZ1239 was isolated from a traveler after visiting Indonesia in 2012. It shares a 98.5% identity with the DH130003 strain [241].
NC/2011–568	HE806461	Asian	The NC/2011–568 was isolated from the sample of the first reported human autochthonous case in New Caledonia in 2011 [242,247].
chikv-sy	KF318729	Asian	The Zhejiang/chik-sy/2012 was the first CHIKV isolate from Zhejiang, China, in 2012. Phylogenetic analysis showed that this strain has a high homology with a strain isolated in Taiwan, which was originally derived from Indonesia, the Indonesia/0706aTw/2007 strain [242,248].
PER160/H803609	KP164571	Asian	These two strains were the first autochthonous CHIKV samples isolated from various parts of Brazil in 2014 [242,249].
AMA2798/H804298	KP164567		
**76 aa duplication event at position 376–456**			
MUM01–2009-Selangor	KX168429	Asian	The MUM01–2009-Selangor was isolated from a DENV2-positive patient sample. It shares a 99% genetic similarity with MY/06/37348 and MY/06/37350 [238].

**Table 2:** List of nsP3 HVD residues and their predicted co-evolved interacting partners, as discovered in silico by Jaspreet et al. in 2016 [242].

nsP3 HVD Residues	Predicted Co-Evolving Partners Residues in nsP3 HVD	Details and Suggested Functions
361D: Located in fragment A. It is also present as a duplicate in the CHIKV MUM01–2009-Selangor strain motif (I) at position 437 (Figure 2b).	408R and 411T: Both are residues located in fragment B (Figure 2a) and at position 477 (Figure 2b).	The 361D residue has 18 co-evolution partners, and 7 of them have the most significant interactions [242]. Both co-evolution interactions between 361D and 408R and 411T are suggested to affect the host cell cycle and modulate the level of proteolysis and peptidolysis activity [242]
464P: Located in fragment C3 (Figure 2a) and at position 533 (Figure 2b).	327S: Located in the *N*-terminal nsP3 HVD and present as a duplicate in the CHIKV MUM01–2009-Selangor strain motif (I) at position 413 (Figure 2b).	The co-evolution is suggested to affect the aa phosphorylation and serine or threonine kinase activity [248,250,251]. The co-evolution interaction is also suggested to be involved in signal transduction—for example, in TGF-β signaling and NF-κB activation [252,253].
	377H: Located in fragment A and present only in CHIKV strains without the 7 aa deletion or 76 aa duplication (Figure 2a).	The 377H and 381S are present in the 4 or 7 aa deleted residues in CHIKV Asian strains. These three co-evolution interactions between 464P and 377H, 381S, and 132M are suggested to be involved in aa phosphorylation and modulate the level of serine or threonine kinase activity [242,254,255].
	381S: Located in fragment A and present only in CHIKV strains without the 4 or 7 aa deletion or 76 aa duplication (Figure 2a).	
	132M: Located in the nsP3 macrodomain	
	395S: Located in fragment B (Figure 2) and at position 464 in the CHIKV MUM01–2009-Selangor strain (Figure 2b).	This interaction was predicted to be involved in the modulation of cell communication, SH3-binding domain, focal adhesion, and virus signal transduction [242].
395S: Located in fragment B (Figure 2) and at position 464 in the CHIKV MUM01–2009-Selangor strain (Figure 2b).	463H: Located in fragment C3 (Figure 2) and at position 532 in the CHIKV MUM01–2009-Selangor strain (Figure 2b).	This interaction was predicted to be involved in the modulation of cell communication, SH3-binding domain, focal adhesion, and virus signal transduction [242].
411T: Located in fragment B (Figure 2) and at position 480 in the CHIKV MUM01–2009-Selangor strain (Figure 2b).	455P: Located in fragment C2 (Figure 2) and at position 524 in the CHIKV MUM01–2009-Selangor strain (Figure 2b).	The interaction is suggested to be involved in receptor signaling, the regulation of circadian rhythm, the response to UV, and protein kinase CK2 activity [242].
	408R: Located in fragment B (Figure 2) and at position 477 in the CHIKV MUM01–2009-Selangor strain (Figure 2b).	This interaction is suggested to be involved in glycosyl group transfer and activation of the MAPK pathway [242].

## Data Availability

Not applicable.
